# Self-generated hydrogel ejects bacterial cells for localized biofilm dispersion

**DOI:** 10.1038/s41564-026-02413-4

**Published:** 2026-07-07

**Authors:** Todd Kwang-Tao Chou, Alejandra Dau-Martinez, Júlia Vicens-Figueres, Arvind Gouttumukkala, Leticia Galera-Laporta, Jordi Garcia-Ojalvo, Gürol M. Süel

**Affiliations:** 1 Department of Molecular Biology, School of Biological Sciences, University of California San Diego, La Jolla, CA USA; 2 Department of Medicine and Life Sciences, Universitat Pompeu Fabra, Barcelona, Spain; 3 Center for Microbiome Innovation, University of California San Diego, La Jolla, CA USA; 4 Synthetic Biology Institute, University of California San Diego, La Jolla, CA USA

**Keywords:** Biofilms, Computer modelling, Microbial communities

## Abstract

Bacteria residing in biofilms are embedded in an extracellular matrix. Whereas biofilm formation is well studied, less is known about biofilm dispersion, although enzymatic extracellular matrix degradation is suspected to play a key role. Here we show that *Bacillus subtilis* biofilms can alternatively eject a specific cell type, locally and anisotropically, using mechanical forces arising from a self-generated hydrogel. Single-cell resolution imaging combined with mathematical modelling, and chemical and genetic perturbations, show that the production of the extracellular poly-γ-glutamic acid (γ-PGA) polymer is necessary to drive this cell ejection. Specifically, osmotic pressure from the γ-PGA hydrogel propels interior cells through the outer layers to break free from the biofilm. We demonstrate control over this process through γ-PGA modulation such that biofilm dispersion can be either inhibited or promoted. Forceful ejection driven by γ-PGA has so far only been described in marine organisms such as jellyfish. Our discovery of biofilm cell ejection via γ-PGA thus reveals not only a previously uncharacterized biofilm dispersion mechanism but also an unexpected mechanistic parallel to evolutionarily distant Cnidaria.

## Abstract (web summary)

*Bacillus subtilis* biofilms locally disperse by ejecting motile cells through osmotic pressure generated by their poly-γ-glutamic acid hydrogel, a mechanism resembling that through which Cnidaria eject nematocysts.

## Main

Cells across all kingdoms of life are often embedded in self-produced extracellular matrix (ECM) networks composed of diverse polymers^1–3^. The ECM holds cells together and provides mechanical support for tissues and cellular communities. Bacterial biofilms are one such biological system where cells live together in a community encased by a self-produced ECM. Nearly all bacteria form biofilms, and *Bacillus subtilis* has served as a robust model system to study biofilm formation for more than 100 years^4^. To form a biofilm, individual bacterial cells produce and secrete an ECM that allows the cells to stick together and to anchor them to surfaces^2^. The ECM offers many benefits for bacterial cells in biofilms, including protection from antibiotics^5–7^. Biofilms thus also constitute a major medical problem, as they can cause chronic infections^8,9^.

**Fig. 1:**
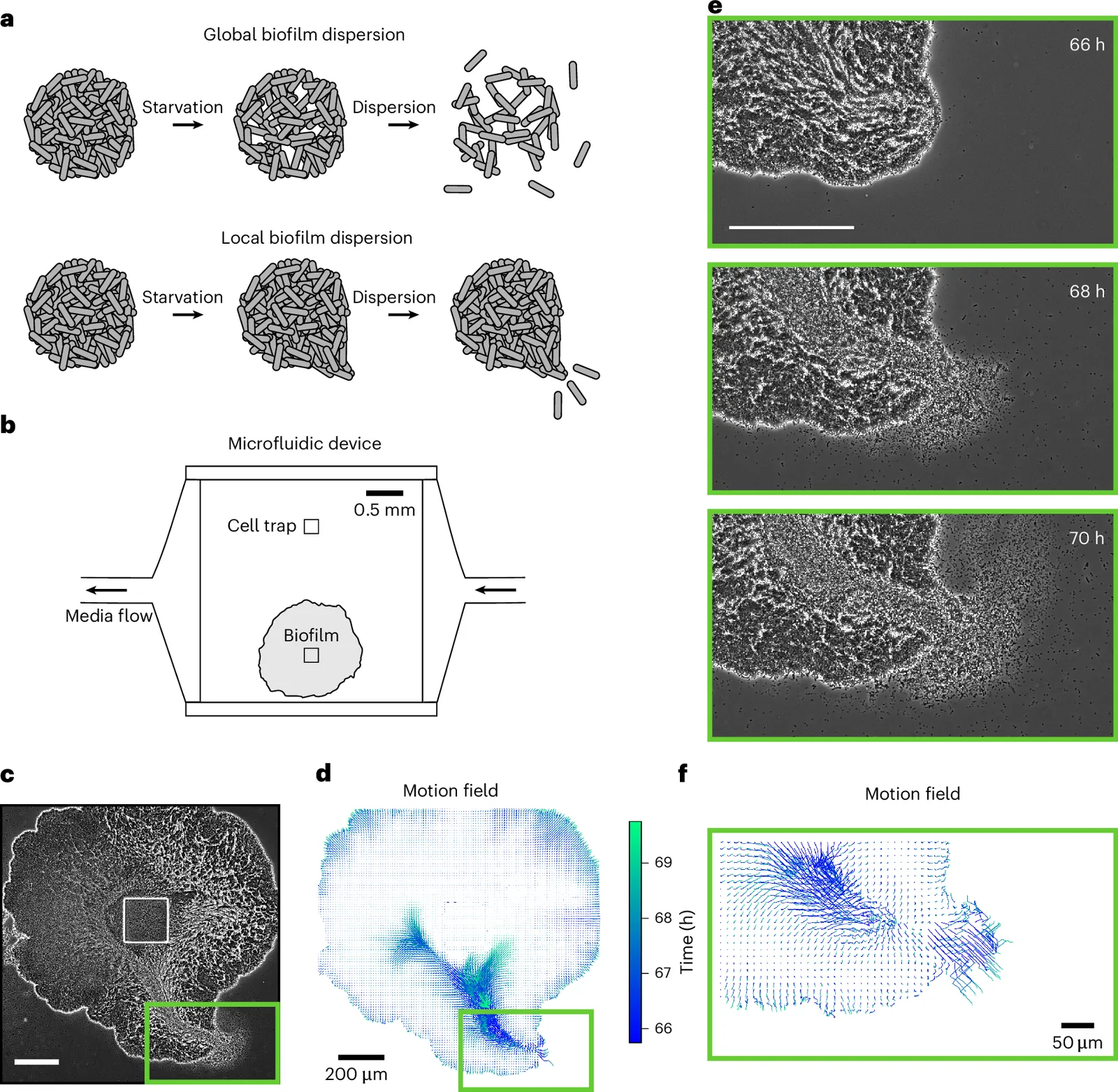
Bacterial biofilms undergo localized dispersion. **a**, When biofilms undergo starvation or encounter other stresses, they disperse in a globally uniform manner (top) by degrading the ECM holding the biofilm together. Cells are known to be spatially organized, but it is unknown whether this spatial heterogeneity can lead to localized biofilm dispersion (bottom). **b**, Schematic of the microfluidic device. Liquid media flow right to left. The growth chamber is 3 mm × 3 mm and contains two cell traps where biofilms can anchor and grow. Cell traps are 200 μm × 200 μm. **c**, Phase-contrast image of a biofilm exhibiting localized dispersion. White square in the middle of the biofilm represents the cell trap. Green rectangle highlights a region of localized dispersion and corresponds to the zoomed-in image shown in **e**. Image shows a representative biofilm from *n* = 3 independent experiments. Scale bar, 200 μm. **d**, Motion field of the biofilm in **c** calculated using Particle Image Velocimetry (Methods). Colours indicate the movement of the biofilm over time. Colour bar is shared between **d** and **f**. **e**, Filmstrip showing the field of view highlighted by the green rectangle in **c**. Images show a representative biofilm from *n* = 3 independent experiments. Scale bar, 100 μm. **f**, Motion field of the zoomed-in region of the biofilm shown in **e**. See also Extended Data Fig. 1 and Supplementary Video 1.

To address these issues, recent studies have investigated possible means to disperse biofilms, thereby allowing for more effective antimicrobial treatments^10–12^. However, in contrast to the detailed understanding of biofilm development, the process of biofilm dispersion is far less understood. According to current understanding, when biofilms undergo nutrient starvation or encounter other stresses, the ECM holding the biofilm together is enzymatically degraded, and cells globally escape the community^10,13–17^. However, it remains unclear whether enzymatically driven ECM degradation is the only mechanism of biofilm dispersion. Furthermore, cell states in biofilms are spatially organized^18–20^, prompting the question of whether such spatial heterogeneity could lead to localized rather than global dispersion (Fig. 1a).

Although biofilm development is initiated by a few cells and can be studied at the single-cell level, dispersion requires imaging of large biofilms over several days. Technical challenges have limited dispersion studies to large biofilms with low spatial resolution^14,21^ or to small colonies of hundreds of cells at short timescales^16,22,23^. Here we used millimetre-scale microfluidic growth chambers to study over several days the dispersion of *B. subtilis* biofilms with more than 1 million cells with near single-cell resolution. We discovered that localized biofilm dispersion is driven by extracellular poly-γ-glutamic acid (γ-PGA) and seems independent of ECM degradation. We demonstrate control over biofilm dispersion using genetic and chemical perturbations. Our results reveal a previously unknown mechanism of biofilm dispersion with intriguing parallels to evolutionarily distant Cnidaria, such as jellyfish, suggesting new approaches to control bacterial community disassembly. The ability to manipulate extracellular hydrogels sets the stage to better understand and control cell release from ECM-embedded cellular communities across the tree of life.

## Results

### Bacterial biofilms undergo localized dispersion

**Extended Data Fig. 1:**
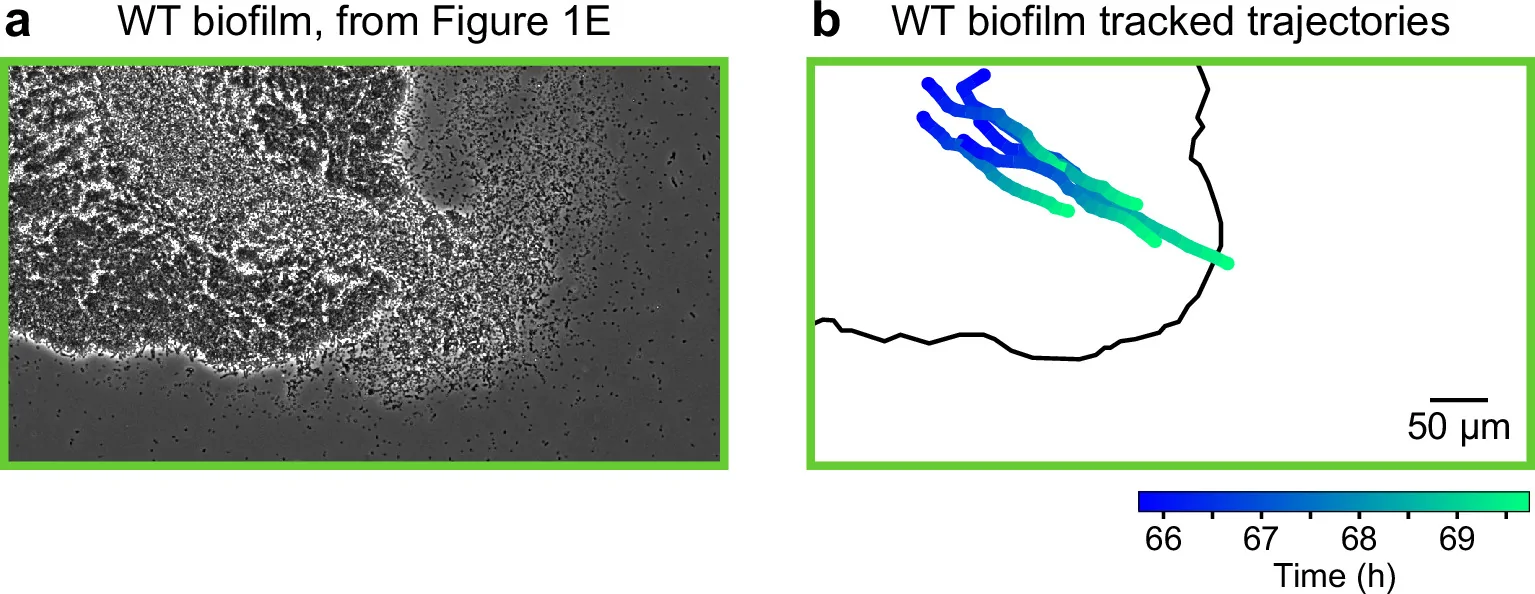
Dispersing cells flow outward. (**A**) Phase contrast image of a dispersing biofilm at 70 h, reproduced from Fig. 1e. (**B**) Five tracked trajectories from the biofilm in (**A**). Time is indicated in blue-to-green color scheme, and the edge of the biofilm is indicated by the black line.

To image the dispersion of large biofilms with more than 1 million cells, we used *B. subtilis* NCIB 3610, a well-characterized undomesticated biofilm-forming strain, culturing it in a microfluidic device with a large growth chamber (3 mm × 3 mm). This chamber had a height of 6 μm, which limited the number of layers of cells in the vertical direction to approximately 10, thus leading to an effectively two-dimensional system (Fig. 1b). The device contained 200 μm × 200 μm cell traps where the biofilm could anchor while the media flowed through the chamber. We used standard MSgg (Minimal Salts, glycerol and glutamate) media, previously reported to promote biofilm formation^24^. Biofilm dispersion is known to be triggered by stress or nutrient starvation^25–27^. Therefore, to trigger dispersion, we first grew biofilms for approximately 30 h until they were established, and then we transiently starved them for 16 h. Starvation was induced by flowing MSgg media without carbon or nitrogen sources (MS). After reintroducing nutrients (standard MSgg), we unexpectedly observed an outward-directed local biofilm dispersion. Rather than the biofilm globally disintegrating, cells escaped the biofilm through what appeared to be a localized ‘river’ (Fig. 1c). To analyse this movement, we used Particle Image Velocimetry and particle tracking (Methods) to determine the flow of cells over time. This analysis showed that dispersion originates from the biofilm interior and flows outwards towards the biofilm edge, without the biofilm globally disintegrating (Fig. 1d). A close-up view of the dispersing area (Fig. 1e,f, Extended Data Fig. 1 and Supplementary Video 1) showed that cells escaped the biofilm in a locally directed manner by flowing out of the biofilm.

**Fig. 2:**
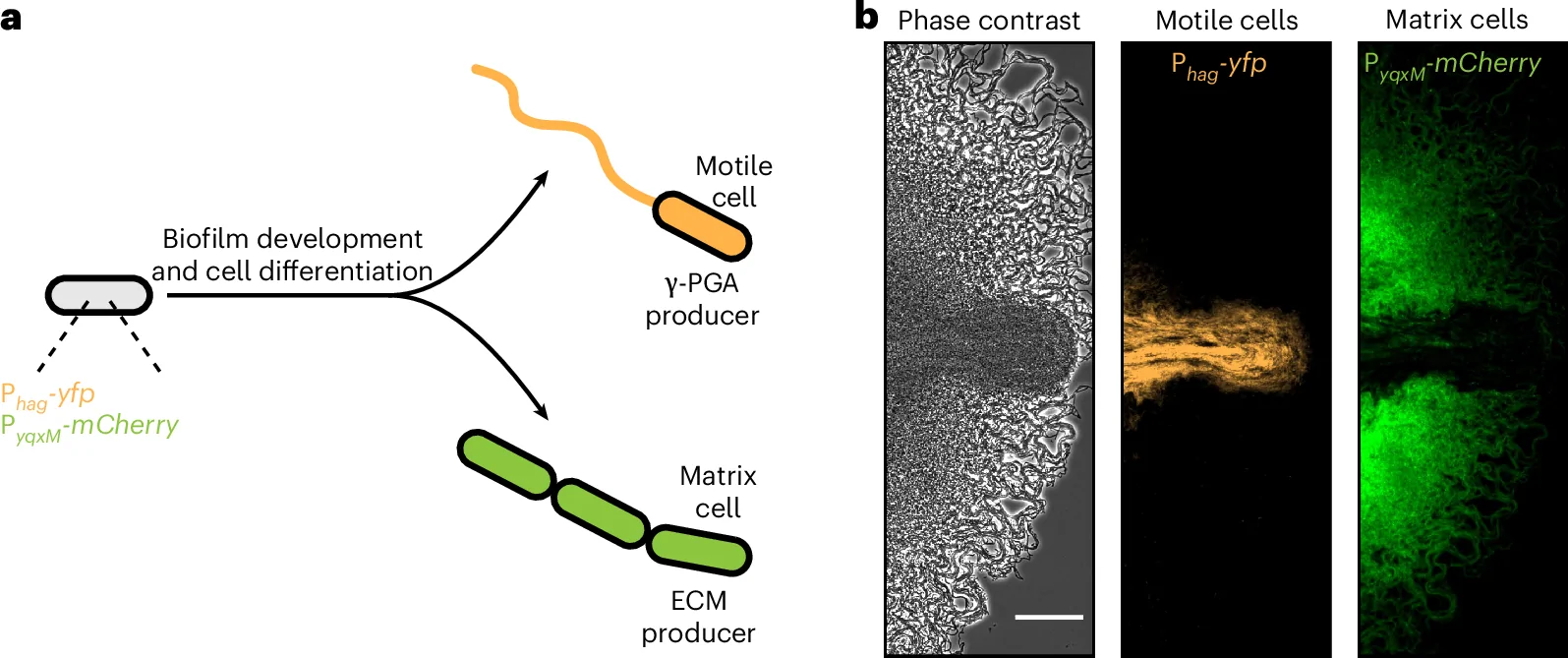
Dispersing region is composed of motile cells. **a**, During biofilm development, cells inside the biofilm differentiate into two mutually exclusive cell types: motile cells and matrix cells. Motile cells are known to produce γ-PGA. Matrix cells are known to produce various ECM components that hold the biofilm together. We monitored the two cell types over space and time using a dual reporter strain: P_*hag*_-*yfp* and P_*yqxM*_-*mCherry*. Motile cells express the fluorescent protein YFP under the *hag* promoter, whereas matrix-producing cells express the fluorescent protein mCherry under the *yqxM* promoter. **b**, Phase-contrast image of the edge of a biofilm (left), fluorescence image of the motile cell reporter P_*hag*_-*yfp* (middle) and fluorescence image of the matrix cell reporter P_*yqxM*_-*mCherry* (right). The dispersing region contains primarily motile cells. Images show a representative biofilm from *n* = 3 independent experiments. Scale bar, 100 μm. See also Extended Data Fig. 2 and Supplementary Video 2.

**Fig. 3:**
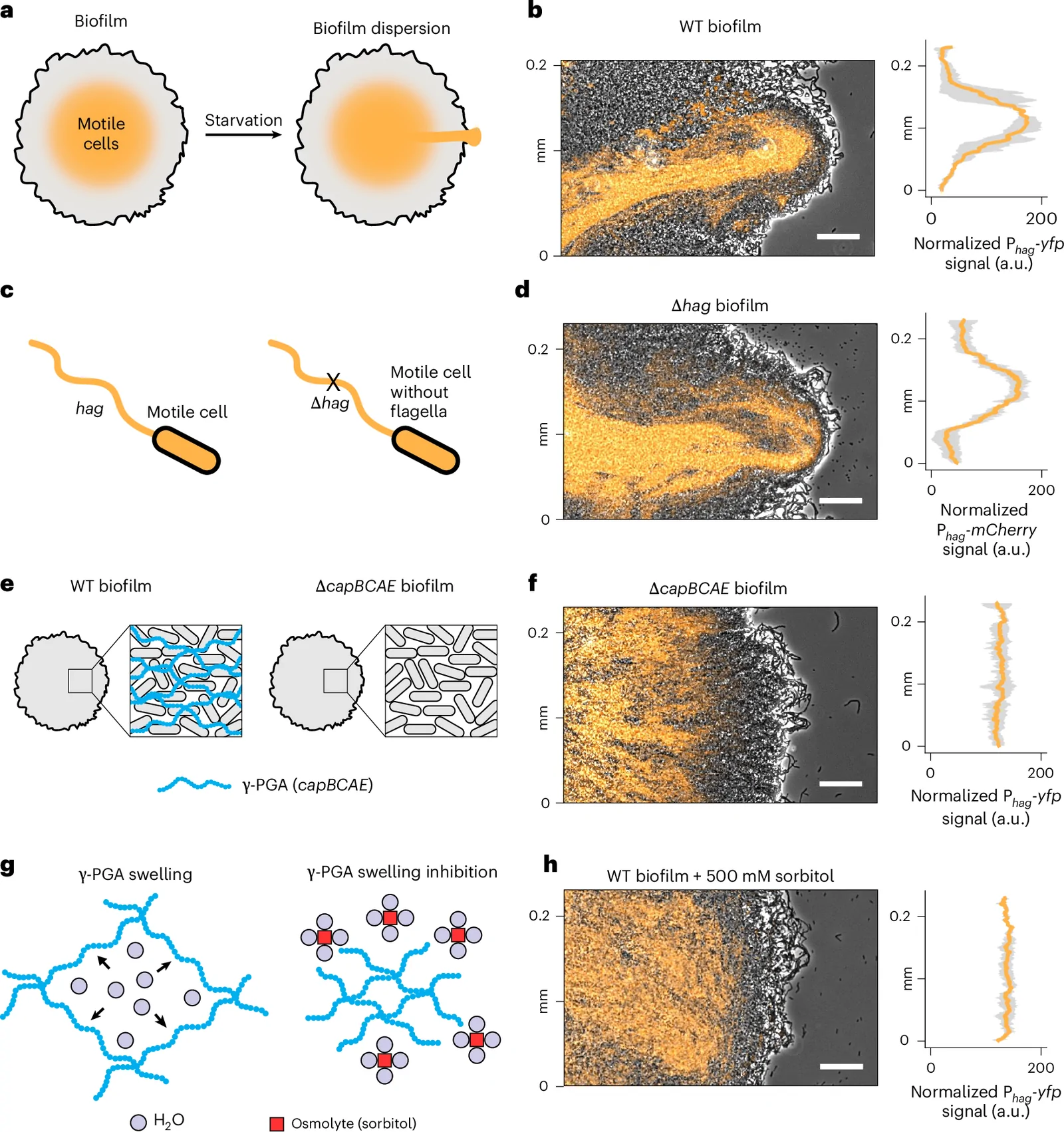
γ-PGA drives localized dispersion without requiring matrix degradation. **a**, Motile cells are known to be located in the centre of the biofilm. Because dispersion also originates from the centre, the dispersing cells should be motile. **b**, Left: phase-contrast image of the edge of a wild-type (WT) biofilm (left). Fluorescence image of the motile cell reporter P_*hag*_-*yfp* is overlaid in orange. P_*hag*_-*yfp* signal is quantified on the right. To compare the P_*hag*_-*yfp* signal across different mutants, we binarized the fluorescence signal and plotted the signal across the image from top to bottom (Methods). Plot shows the average for *n* = 3 dispersion events from independent biofilms. Grey-shaded region represents the standard deviation. Scale bar, 50 μm. **c**, The *hag* gene encodes for flagellin, which makes up the flagella. We deleted this gene to investigate whether motility is required for dispersion. **d**, Left: phase-contrast image of the edge of a *hag* deletion biofilm, with P_*hag*_-*mCherry* overlaid in orange. Right: P_*hag*_-*mCherry* signal is quantified in the same manner as in **b**. Plot shows *n* = 3 independent biofilms. Shaded region indicates standard deviation. Scale bar, 50 μm. **e**, If cells are not using flagella to swim out of the biofilm, how are they dispersing? As motile cells are also known to produce and excrete γ-PGA (indicated in blue), we deleted the operon responsible for its production (*capBCAE*). **f**, Left: phase-contrast image of the edge of a *capBCAE* deletion biofilm, with P_*hag*_-*yfp* overlaid in orange. Right: P_*hag*_-*yfp* signal is quantified in the same manner as in **b**. Plot shows *n* = 3 independent biofilms. Shaded region indicates standard deviation. Scale bar, 50 μm. **g**, γ-PGA is known to absorb water. To test whether this swelling is responsible for dispersion, we added 500 mM of the charge-neutral osmolyte sorbitol into the growth medium, which will prevent γ-PGA from swelling. **h**, Left: phase-contrast image of the edge of a wild-type biofilm grown with 500 mM sorbitol, with P_*hag*_-*yfp* overlaid in orange. Right: P_*hag*_-*yfp* signal is quantified in the same manner as in **b**. Plot shows *n* = 3 independent biofilms. Shaded region indicates standard deviation. Scale bar, 50 μm. See also Extended Data Figs. 3–6.

**Extended Data Fig. 2:**
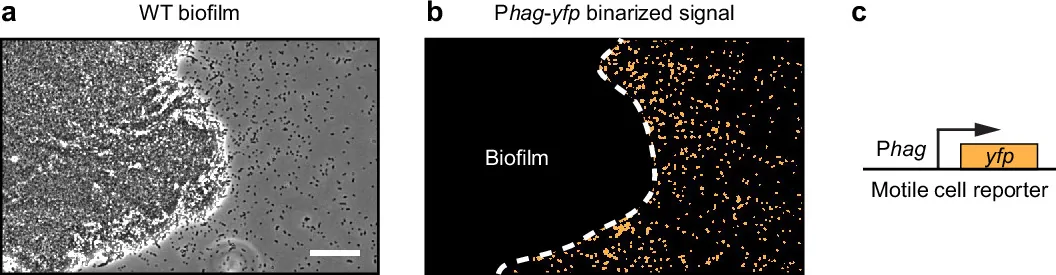
Dispersed cells are motile. (**A**) Phase contrast image of a biofilm undergoing localized dispersion. Image shows a representative biofilm from n = 3 independent experiments. Scale bar, 50 μm. (**B**) Fluorescence image of the same region shown in (**A**). P_*hag*_-*yfp* fluorescence is colored in orange. Fluorescence from the biofilm is removed to highlight already dispersed cells. The edge of the biofilm is indicated as a white dashed line, determined using the phase contrast image. The region outside the biofilm was binarized to enhance the motile cell signal, which is low due to the low cell density. (**C**) P_*hag*_-*yfp* is a fluorescent reporter of motile cells. Yellow fluorescent protein (*yfp*) is expressed under the control of the natural *hag* gene promoter (P_*hag*_). This promoter activates flagellin production only in motile cells.

**Extended Data Fig. 3:**
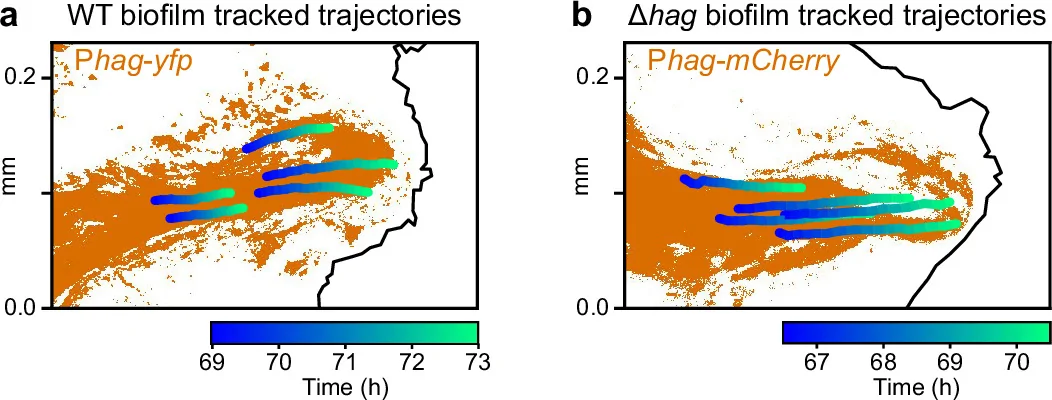
Dispersing motile cells flow outward. (**A**) Five tracked trajectories in the wild-type biofilm from Fig. 3b. Time is indicated in blue-to-green color scheme, and the edge of the biofilm is indicated by the black line. Region of P*hag*-*yfp* expression is indicated in orange. (**B**) Five tracked trajectories in the Δ*hag* biofilm from Fig. 3d. Time is indicated in blue-to-green color scheme, and the edge of the biofilm is indicated by the black line. Region of P*hag*-*mCherry* expression is indicated in orange.

Given the anisotropic nature of biofilm dispersion, we asked whether a specific cell type, heterogeneously distributed through the biofilm, was responsible for dispersion. Bacterial biofilms are composed of two major and mutually exclusive cell types, motile and matrix cells^28,29^ (Fig. 2a). We used a dual reporter strain to track both motile and matrix cell types in the biofilm^30,31^. Specifically, we tracked matrix cells using the transcriptional fluorescent reporter P_*yqxM*_-*mCherry*, and motile cells using the transcriptional fluorescent reporter P_*hag*_-*yfp*. Using this dual reporter strain, we determined that the dispersing regions were primarily composed of motile cells (Fig. 2b and Supplementary Video 2). We also confirmed that the cells that escaped the biofilm were indeed motile (Extended Data Fig. 2). Consistent with previous work indicating that motile cells are located in the biofilm interior^32,33^, dispersing motile cells originated from the biofilm interior and were pushed outwards (Fig. 3a,b and Extended Data Fig. 3).

Previous work has shown that motility is an important contributor to dispersion^16,34^. Therefore, we tested whether cell motility is responsible for the localized dispersion phenomenon that we observed. Accordingly, we constructed a mutant strain that lacks the *hag* gene and cannot make flagella (Δ*hag*; Fig. 3c). The Δ*hag* strain only deletes the downstream structural component of flagella but does not prevent cells from residing in the overall ‘motile state’ that is defined by upstream transcriptional regulators. Using the same transcriptional fluorescent reporter for the motile state, we found that biofilms formed by the Δ*hag* strain still exhibited dispersion in the same manner as the wild-type strain (Fig. 3d). This unexpected result indicates that although the motility cell type correlates with the dispersing regions, cells do not require active swimming to break free from the biofilm.

### γ-PGA drives localized biofilm dispersion

**Extended Data Fig. 4:**
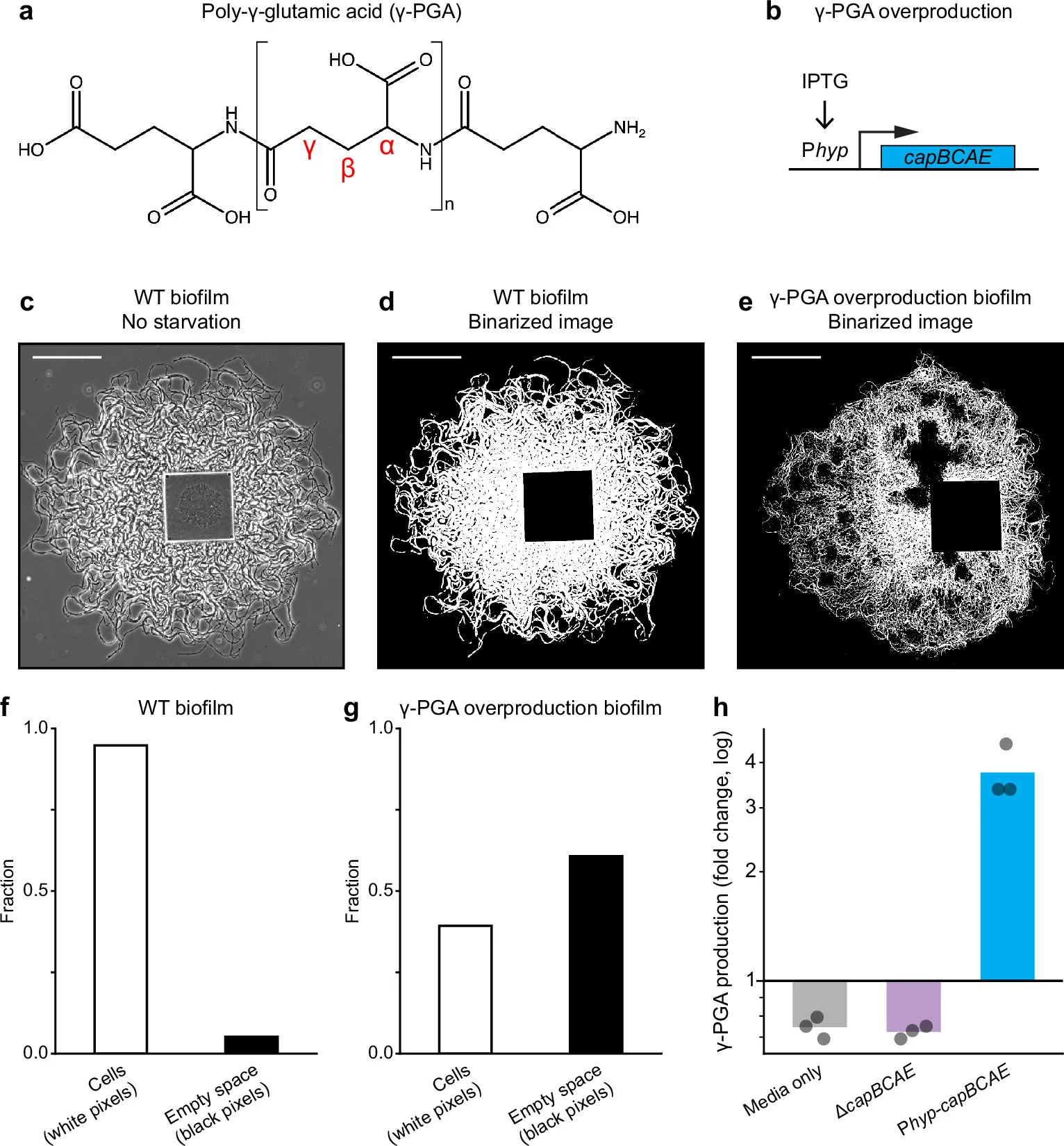
Poly-γ-glutamic acid overproduction strain. (**A**) Poly-γ-glutamic acid (γ-PGA) is a polymer composed of L-glutamic acid and D-glutamic acid monomers linked together by γ-amino bonds, as opposed to most proteins which are linked together by α-amino bonds. The α, β, and γ carbons of each glutamic acid monomer are indicated in red. (**B**) Schematic of the γ-PGA overproduction construct. The operon responsible for γ-PGA production (*capBCAE*) was placed under the control of the inducible *hyperspank* promoter. This promoter is fully induced with 1 mM IPTG. (**C**) Phase contrast image of a wild-type biofilm grown without starvation to prevent triggering natural dispersion. Media contained 30 mM D-glutamic acid and 1 mM IPTG to compare with the γ-PGA overproduction strain. Image shows a representative biofilm from n = 3 independent experiments. Scale bar, 200 μm. (**D**) Image from (**C**) was binarized to emphasize areas where cells are present or absent. Image shows a representative biofilm from n = 3 independent experiments. Scale bar, 200 μm. (**E**) Phase contrast image of a γ-PGA overproduction biofilm after 21 h of growth without starvation to prevent triggering natural dispersion was binarized as in (**D**). Media contained 30 mM D-glutamic acid and 1 mM IPTG to provide substrate for γ-PGA production. Image shows a representative biofilm from n = 3 independent experiments. Scale bar, 200 μm. (**F**) Quantification of the fraction of white pixels (cells are present) or black pixels (cells are absent) of the image in (**D**). The area outside of the biofilm and the area under the cell trap are excluded from analysis. (**G**) Quantification of the fraction of white and black pixels of the image in (E) performed in the same manner as (**F**). (**H**) Quantification of γ-PGA production relative to wild-type measured by methylene blue (see Methods). n = 3 for each condition.

**Extended Data Fig. 5:**
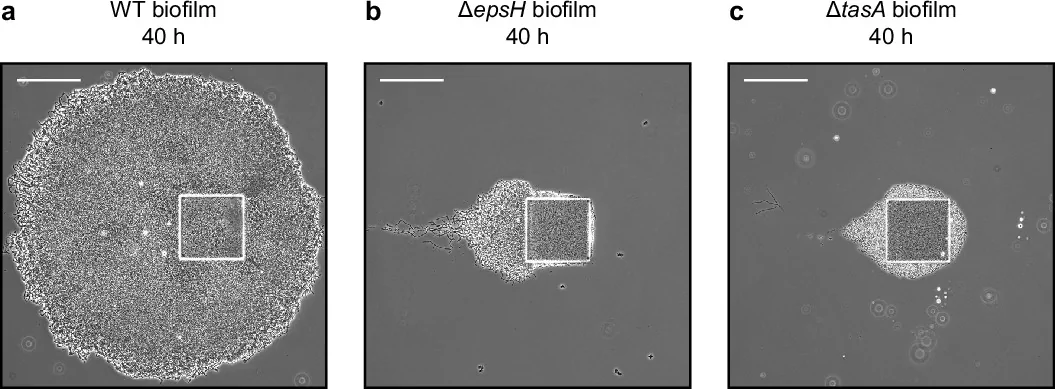
Biofilms without matrix components fail to grow. (**A**) Phase contrast image of a wild-type biofilm grown in standard MSgg for 40 h. Image shows a representative biofilm from n = 3 independent experiments. Scale bar, 200 μm. (**B**) Phase contrast image of an Δ*epsH* biofilm grown in standard MSgg for 40 h. *epsH* encodes for exopolysaccharides, a component of the biofilm extracellular matrix. Without *epsH*, cells in the biofilm are not strongly held together, and cells are washed out due to media flow. Image shows a representative biofilm from n = 3 independent experiments. Scale bar, 200 μm. (**C**) Phase contrast image of a Δ*tasA* biofilm grown in standard MSgg for 40 h. *tasA* encodes for amyloid fibers, a component of the biofilm extracellular matrix. Without *tasA*, cells in the biofilm are not strongly held together, and cells are washed out due to media flow. Image shows a representative biofilm from n = 3 independent experiments. Scale bar, 200 μm.

**Extended Data Fig. 6:**
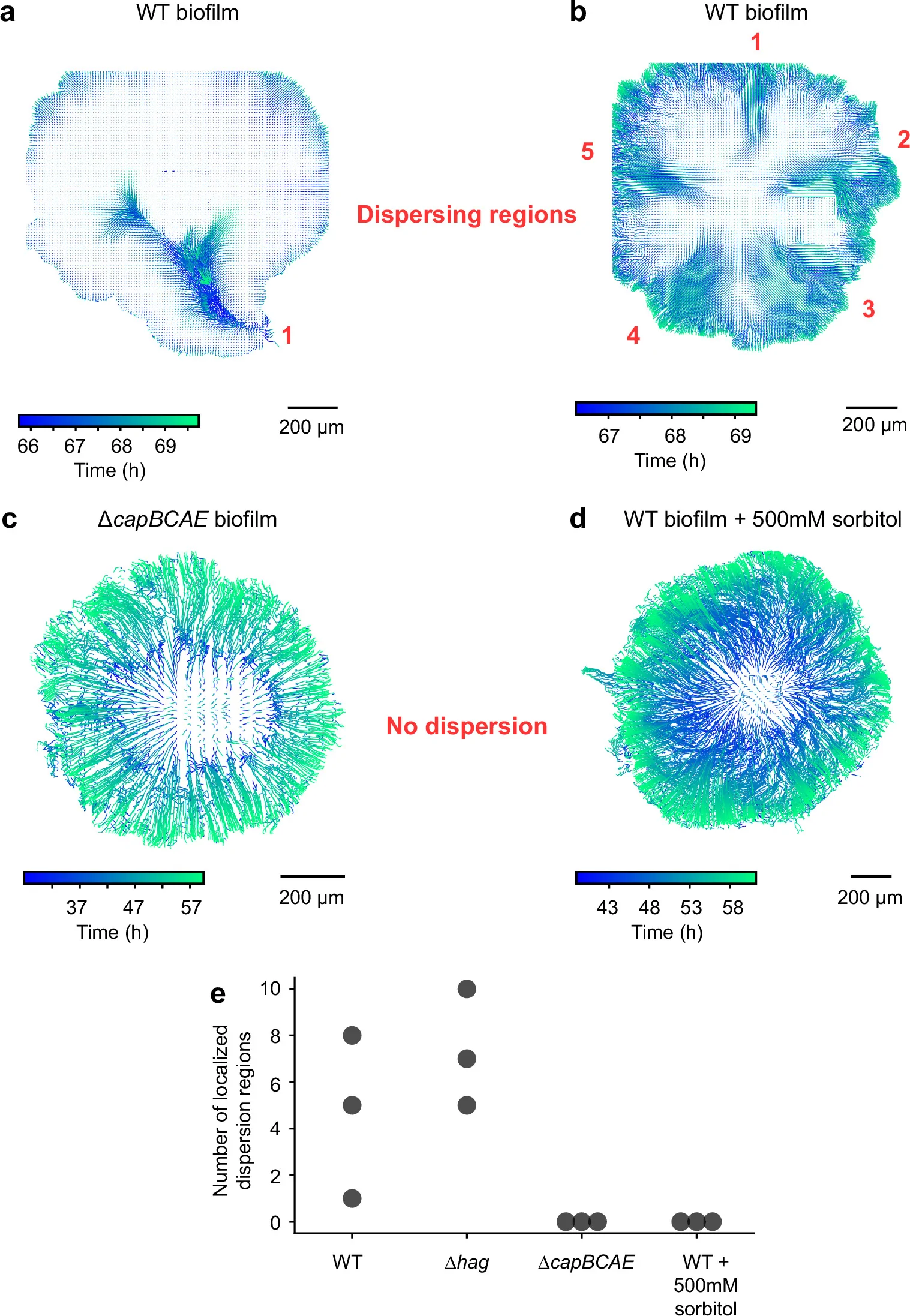
Biofilm dispersion is inhibited by γ-PGA deletion or sorbitol addition. (**A**) Motion field of a wild-type biofilm, reproduced from Fig. 1d. Colors indicate the movement of the biofilm over time. Scale bar, 200 μm. (**B**) Motion field of a second wild-type biofilm showing five dispersion regions. Scale bar, 200 μm. (**C**) Motion field of a Δ*capBCAE* biofilm. Scale bar, 200 μm. (**D**) Motion field of a wild-type biofilm with 500 mM of sorbitol added to the growth medium. Sorbitol is a charge-neutral osmolyte. Scale bar, 200 μm. (**E**) Quantification of how many localized regions of dispersion each biofilm exhibits for wild-type, Δ*hag*, Δ*capBCAE*, and wild-type with sorbitol biofilms. n = 3 biofilms for each condition.

Given that the motility cell state correlates with localized dispersion, but active swimming is not required, what then is the underlying driver? Cells in the motility state express genes other than those required for swimming. In particular, motile cells produce the extracellular γ-PGA^35,36^. This is particularly interesting because previous work has shown that Cnidaria such as jellyfish and hydra use γ-PGA to eject their stinging threads^37,38^. We therefore hypothesized that bacterial biofilms may use γ-PGA in a similar manner to eject cells from the community. To investigate whether γ-PGA production is indeed responsible for localized biofilm dispersion, we deleted the operon responsible for its production, *capBCAE*^39,40^ (also called *pgsBCAE*) (Fig. 3e). We confirmed that this strain did not produce γ-PGA (Extended Data Fig. 4). This γ-PGA-deficient strain still formed full-size biofilms in the microfluidic device, which is in contrast to strains with a deletion for the major ECM components^24,30,41^ (exopolysaccharides or amyloid fibres) that failed to form biofilms (Extended Data Fig. 5). Importantly, biofilms formed by the γ-PGA-deficient strain did not exhibit dispersion (Fig. 3f and Extended Data Fig. 6). Given that genes related to ECM degradation were still present in this γ-PGA-deficient strain, ECM degradation does not seem to be critical to drive the observed localized dispersion. We thus find that localized biofilm dispersion requires γ-PGA production.

**Extended Data Fig. 7:**
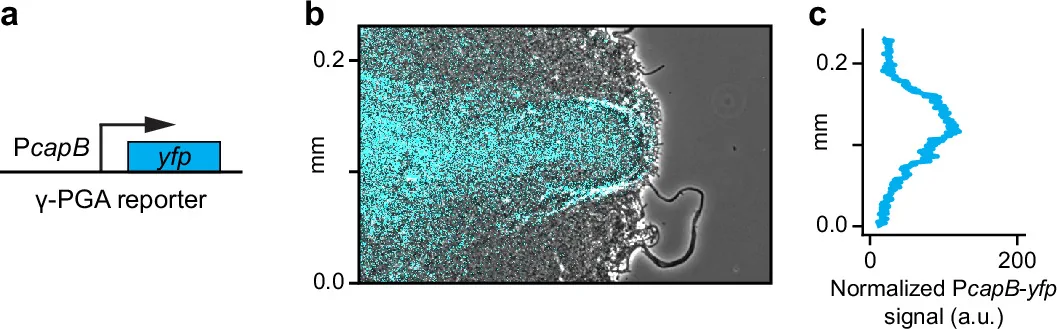
γ-PGA is expressed in the dispersing regions. (**A**) Diagram of the P_*capB*_-*yfp* construct that reports on γ-PGA production. Yellow fluorescent protein (*yfp*) is expressed under the control of the promoter of the operon responsible for γ-PGA production (*capBCAE*). (**B**) Phase-contrast image of a wild-type biofilm with the P_*capB*_-*yfp* construct. Fluorescence image of the γ-PGA production reporter is overlaid in cyan. (**C**) Quantification of P_*capB*_-*yfp* signal performed in the same manner as in Fig. 3 (also see Methods).

To confirm that γ-PGA is necessary to induce localized dispersion, we constructed a fluorescent reporter for γ-PGA expression (P_*capB*_-*yfp*) and found that the dispersing regions express γ-PGA production genes (Extended Data Fig. 7). Next, we focused on the known hydrogel properties of γ-PGA. Work on Cnidaria has shown that γ-PGA is a hydrogel that can swell and generate osmotic pressure^37,38^. We hypothesized that if we introduced an osmolyte into our media, it would compete against biofilm γ-PGA for water, thereby preventing γ-PGA swelling and inhibiting dispersion (Fig. 3g). To test this hypothesis, we introduced 500 mM of the charge-neutral osmolyte sorbitol to wild-type biofilms and then observed that dispersion was inhibited (Fig. 3h and Extended Data Fig. 6). This result indicates that simply adding a charge-neutral osmolyte that competes for water to the growth medium can inhibit dispersion, supporting the hypothesis that osmotic swelling of γ-PGA drives localized dispersion.

### γ-PGA swelling pushes cells apart

**Fig. 4:**
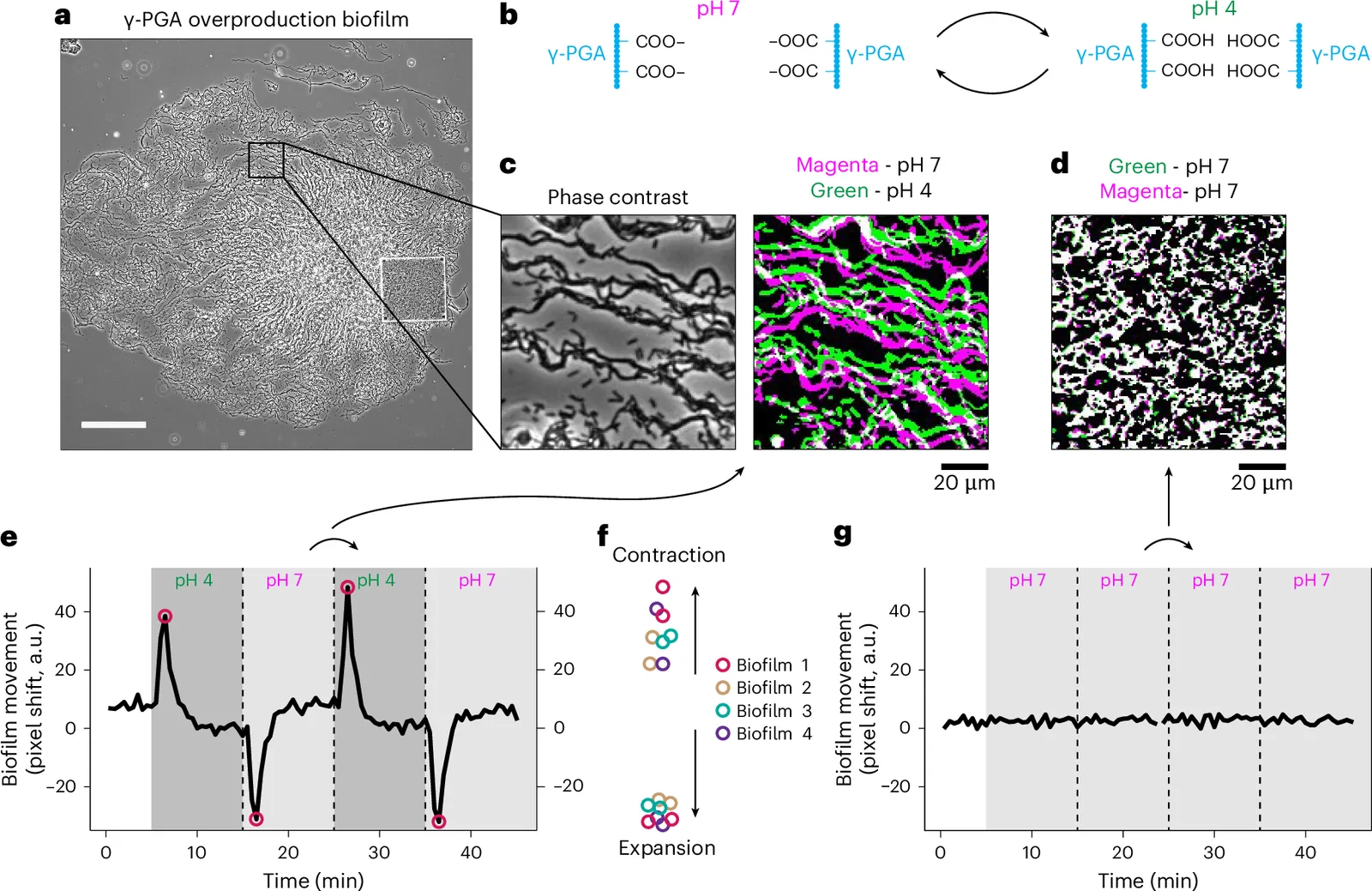
γ-PGA swelling pushes cells apart. **a**, Phase-contrast image of a γ-PGA overproduction biofilm after 21 h (P_*hyp*_-*capBCAE*). Image shows a representative biofilm from *n* = 3 independent experiments. Scale bar, 200 μm. **b**, Carboxyl groups of the γ-PGA polymer are deprotonated at pH 7, so the polymers push apart owing to electrostatic repulsion. Carboxyl groups of the γ-PGA polymer are protonated at pH 4, so the polymers can be tightly packed. We used the microfluidic device to alternate the growth media between pH 7 and 4. **c**, Left: zoomed-in phase-contrast image of the γ-PGA overproduction biofilm from **a**. Right: the phase image of the zoomed-in region exposed to pH 7 is in magenta, whereas the phase image of the region exposed to pH 4 is in green. These images are overlaid to show the shift in cell location owing to the media change. Images show a representative biofilm from *n* = 3 independent experiments. **d**, To ensure that the shift in cell location was not due to media switching, we flowed in new media at the same intervals as in **c**, but the pH remained at 7. Image shows a representative biofilm from *n* = 3 independent experiments. **e**, Successive phase-contrast images of the entire biofilm in **c** were subtracted from one time point to the next. Positive values indicate biofilm contraction. Negative values indicate biofilm expansion. Light grey-shaded regions correspond to media at pH 7, and dark grey-shaded regions correspond to media at pH 4. Maximum and minimum values are indicated by red dots. **f**, Maximum and minimum values from *n* = 4 independent biofilms are shown, with each biofilm represented in a different colour. **g**, The entire biofilm from **d** was analysed in the same manner as in **e**. See also Extended Data Fig. 4.

To further investigate the ability of γ-PGA expression and swelling to push cells apart, we constructed a γ-PGA-overproducing strain (Extended Data Fig. 4). We used the isopropyl-β-d-1-thiogalactopyranoside (IPTG)-inducible *hyperspank* promoter to drive the expression of the *capBCAE* operon, which encodes enzymes for γ-PGA biosynthesis. This engineered strain produces almost fourfold more γ-PGA than the wild type (Extended Data Fig. 4). We then investigated biofilms formed by this γ-PGA-overproducing strain. To avoid triggering natural biofilm dispersion, we purposefully grew both wild-type and γ-PGA-overproducing biofilms without exposing them to nutrient starvation. To ensure that γ-PGA overproduction was not substrate-limited, we supplemented both the wild-type and γ-PGA-overproducing strains with d-glutamic acid (30 mM; Methods). The continuous supply of media with additional d-glutamic acid did not trigger dispersion in wild-type biofilms (Extended Data Fig. 4). By contrast, under the same conditions, the γ-PGA-overproducing biofilm exhibited voids that appeared to arise when cells were being pushed apart (Fig. 4a and Extended Data Fig. 4). To quantify the biofilm density of wild-type and γ-PGA-overproducing phenotypes, we binarized the phase-contrast images and identified areas where cells were present (white pixels) and areas devoid of cells (black pixels). The results showed that the γ-PGA overproduction biofilm had far more regions devoid of cells than the wild-type biofilm (Extended Data Fig. 4). Increased expression of γ-PGA and subsequent swelling thus create biofilms with detectable voids throughout.

To test whether the gaps between cells observed in the γ-PGA-overproducing biofilm were indeed due to the hydrogel properties of γ-PGA, we performed another experiment inspired by work on Cnidaria. Specifically, Cnidaria produce γ-PGA in an acidic nematocyst, where the carboxyl groups of γ-PGA (Extended Data Fig. 4) are protonated, enabling tight packing. As water enters and deacidifies the nematocyst, γ-PGA carboxyl groups become deprotonated, exposing negative charges that drive electrostatic repulsion and separate the polymers^42^. We thus predicted that biofilms could be dynamically expanded or contracted by altering the pH of the growth medium (Fig. 4b).

To test our prediction, we switched the growth media between pH 7 and pH 4 every 10 min and imaged the voids in the γ-PGA-overproducing biofilm. We performed this pH change several times per biofilm. We colour-coded the bacterial cells according to media pH to visualize the expansion and contraction of the spaces between cells (Fig. 4c). We indeed observed the expected expansion at neutral pH and the contraction at acidic pH. We show that biofilm expansion and contraction are not due to media switching but require a change in pH (Fig. 4d). These data are consistent with the predicted change in the protonation state of γ-PGA. We also quantified the biofilm contraction and expansion by systematically subtracting successive images from one time point to the next. The pixel shift of successive images confirms that the biofilm expanded when the pH changed from 4 to 7, and contracted when the pH changed from 7 to 4 (Fig. 4e,f). Switching the media without changing the pH had no such effect (Fig. 4g). Together, these data show that the spacing of cells in the biofilm can be dynamically controlled through pH modulation, consistent with the Cnidaria literature and the electrochemical properties of the γ-PGA polymer.

### Mathematical modelling predicts biofilm break-up

**Fig. 5:**
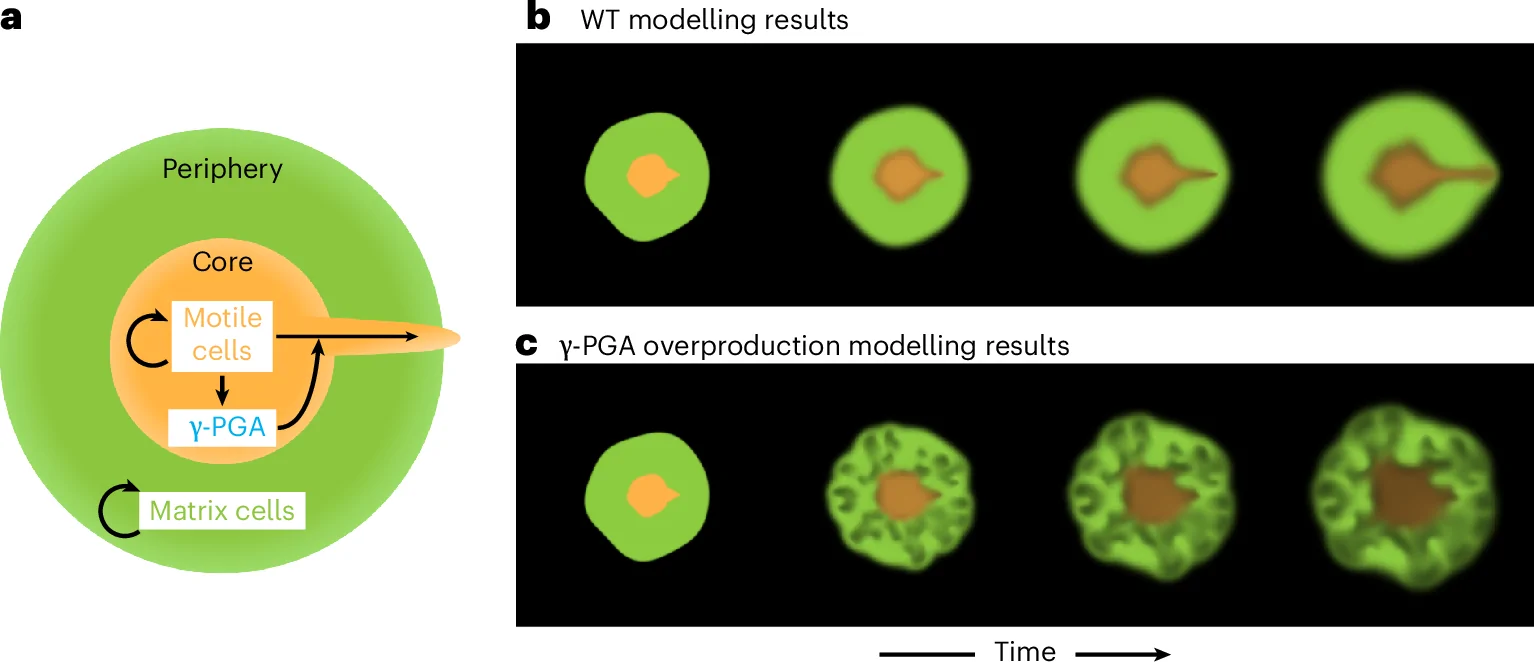
Mathematical modelling reproduces dispersion and predicts biofilm break-up. **a**, Schematic of the modelling strategy. Non-motile and motile bacterial cells are modelled as continuum density fields. Motile cells produce γ-PGA, represented by another continuum field. The resulting γ-PGA hydrogel swells, leading to the dispersion of the motile population. Only the main interactions between the three fields are shown here. A fourth field, representing nutrients (not shown in the scheme), is also considered to modulate cell growth and γ-PGA production, as discussed in detail in the Methods. **b**, Filmstrip showing the dynamics of a wild-type biofilm undergoing localized dispersion driven by pushing. The first snapshot shows the initial condition of the model simulation. **c**, Filmstrip showing the dynamics of a γ-PGA-overproducing biofilm undergoing global breakdown driven by widespread dispersion. The first snapshot shows the initial condition of the model simulation. See also Extended Data Fig. 8 and Supplementary Videos 3 and 4.

**Extended Data Fig. 8:**
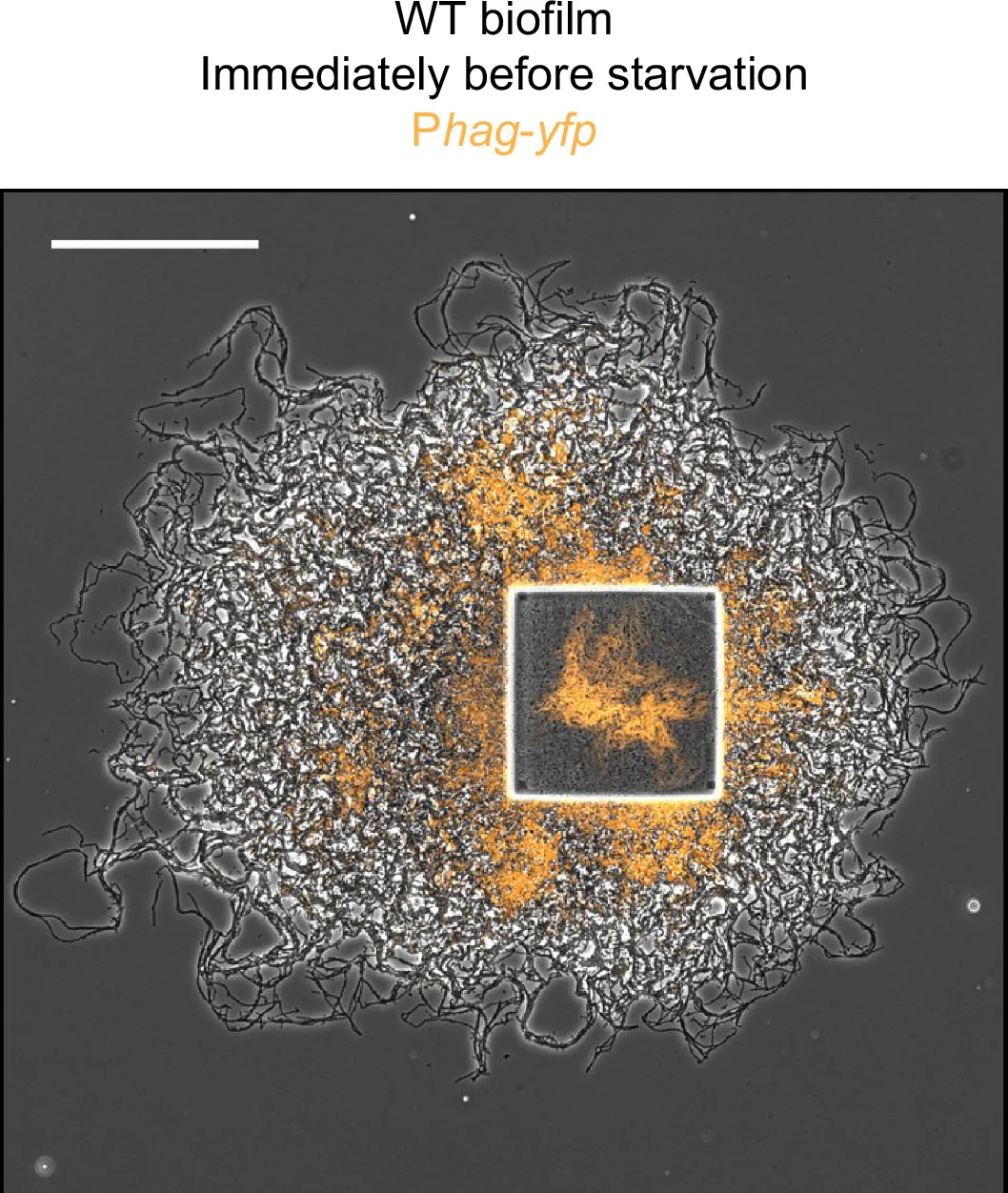
Motile cells are localized to the biofilm interior. A wild-type biofilm imaged just before the starvation protocol to trigger dispersion. The fluorescence image of the motile cell reporter P_*hag*_-*yfp* is overlaid in orange. Image shows a representative biofilm from n = 3 independent experiments. Scale bar, 200 μm.

We turned to modelling to further validate that γ-PGA swelling is sufficient to produce localized dispersion in biofilms and to generate testable predictions. Specifically, we developed a continuous model to capture the mechanical effects of bacterial cells^43^, together with the production and swelling of γ-PGA (Methods). The model includes both matrix and motile cell types, with the latter preferentially located in the centre of the biofilm, consistent with our observations and with the literature^32,33^ (Fig. 5a; see also Fig. 3a). Guided by the literature^44^ and our observations (Extended Data Fig. 8), we started our simulations with a non-isotropic motile cell density distribution (Methods). γ-PGA swells upon being secreted, which (1) generates mechanical pressure on the motile cells, enabling them to move outward, and (2) locally increases cell mobility with respect to the neighbouring densely packed areas of the biofilm (Methods). As the γ-PGA-rich region expands, it effectively behaves as a fluid invading a more viscous medium. This viscosity difference, together with the pressure gradient, recapitulates the localized and outward-directed dispersion phenotype observed experimentally (Fig. 5b and Supplementary Video 3).

We then used the model to determine the impact of γ-PGA overproduction on biofilm dispersion. Such overexpression would be expected to increase the movement of cells more broadly in the biofilm. Our simulations show that increased γ-PGA production results in widespread dispersion that leads to the breakdown of the population into clusters of cells, thus compromising the biofilm integrity (Fig. 5c and Supplementary Video 4).

### γ-PGA overproduction forces biofilms to break apart

**Fig. 6:**
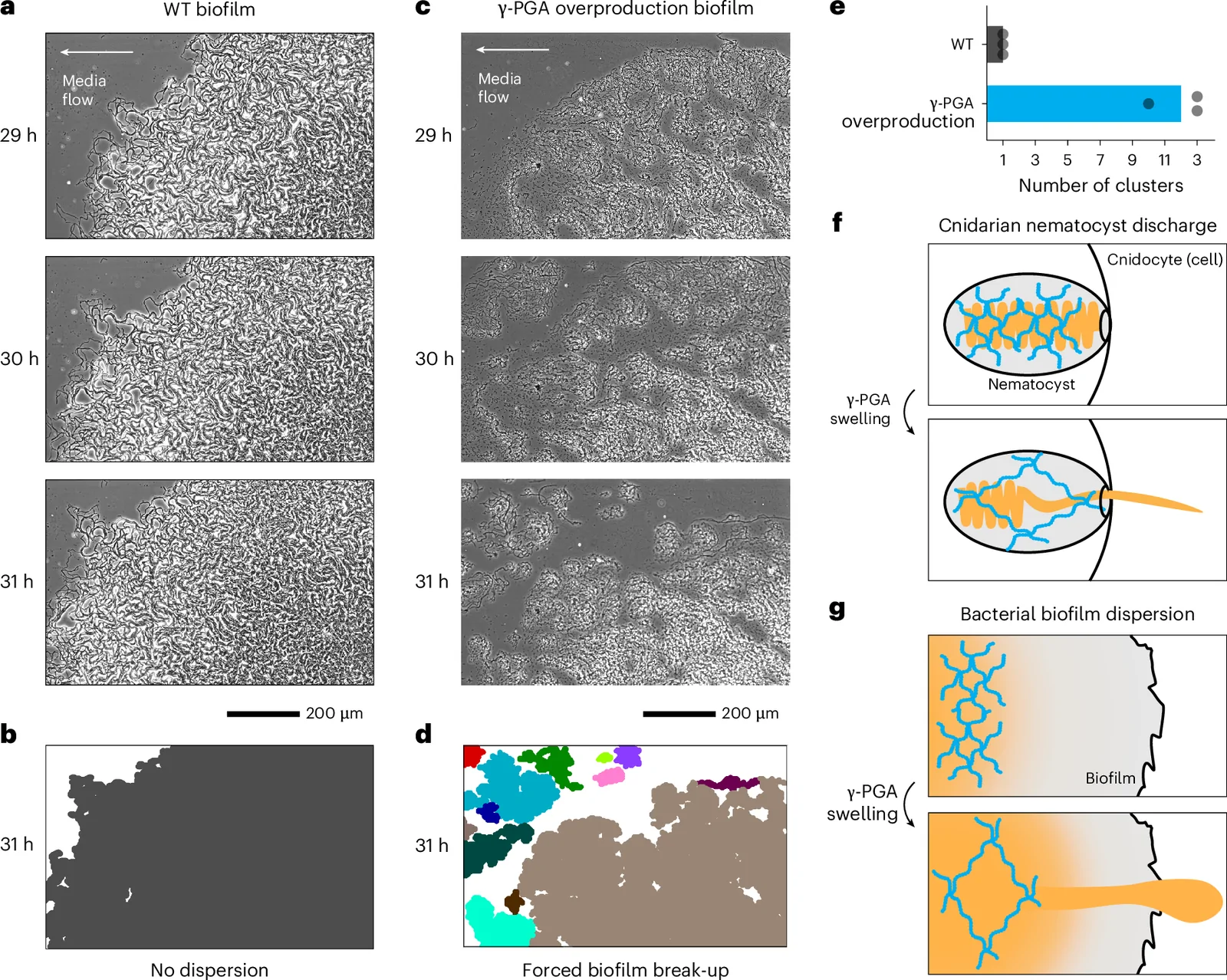
γ-PGA overproduction forces biofilms to break apart. **a**, Filmstrip of phase-contrast images of a wild-type biofilm grown without starvation to avoid triggering natural biofilm dispersion. Direction of media flow in the microfluidic device is indicated by the white arrow. **b**, Cluster analysis of the phase-contrast image of the wild-type biofilm at 31 h (Methods). The biofilm grows as one continuous cluster. **c**, Filmstrip of phase-contrast images of a γ-PGA overproduction biofilm grown without starvation to avoid triggering natural biofilm dispersion. The biofilm grows and breaks apart. Broken-off cell clusters are washed away from the main biofilm owing to media flow. Direction of media flow in the microfluidic device is indicated by the white arrow. **d**, Cluster analysis of the phase-contrast image of the γ-PGA overproduction biofilm at 31 h (Methods). There are 13 disconnected clusters, each represented in a different colour. **e**, Quantification of the number of clusters observed for wild-type and γ-PGA overproduction biofilms. Cluster analysis of *n* = 3 independent biofilms for each condition. Bars show the mean value, and each dot represents a separate biofilm. **f**, Nematocysts are cells that store the stinging thread for Cnidaria such as jellyfish and hydra. They produce γ-PGA inside an acidic environment. When water enters the compartment, the γ-PGA swells and provides osmotic pressure to eject the stinging thread. Furthermore, the γ-PGA polymers become deprotonated and further push the stinging thread out of the compartment through electrostatic repulsion. **g**, Biofilms produce γ-PGA to disperse. γ-PGA absorbs water to generate osmotic force. Furthermore, individual γ-PGA polymers push apart from each other owing to electrostatic repulsion. This process is mechanistically and functionally similar to cnidarian nematocyst discharge. See also Extended Data Fig. 9 and Supplementary Video 5.

**Extended Data Fig. 9:**
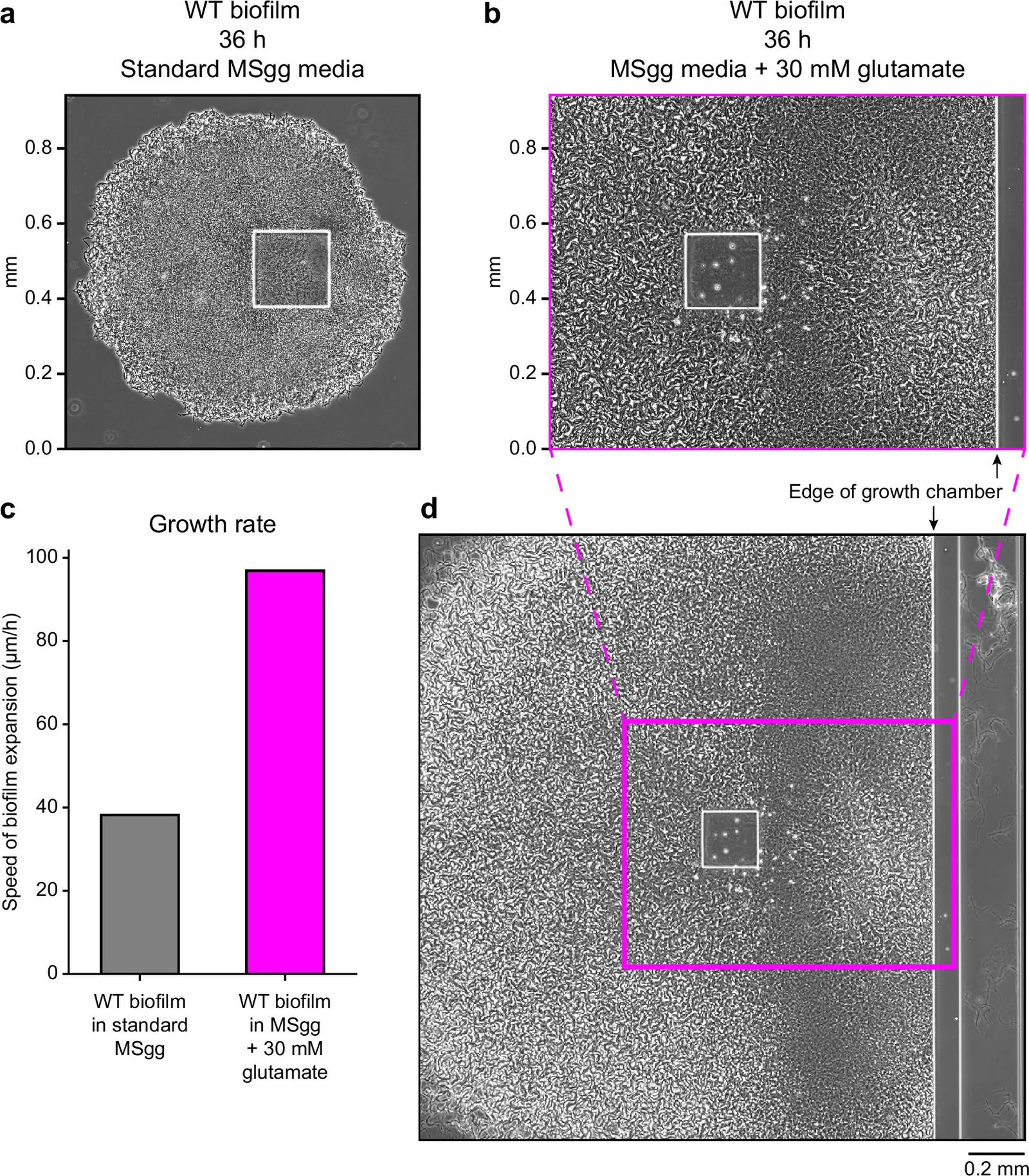
Glutamate addition results in increased growth rather than biofilm breakup. (**A**) Phase contrast image at 36 h of a wild-type biofilm growing in standard MSgg media. (**B**) Phase contrast image at 36 h of a wild-type biofilm growing in MSgg media supplemented with 30 mM glutamate. (**C**) Growth rate of biofilms shown in (**A**) and (**B**) measured by how fast the biofilm radius increases over time (μm/h). (**D**) Phase-contrast image of the same biofilm as (**B**). Four fields of view are stitched together to show that addition of glutamate leads to high biofilm growth without inducing biofilm breakup.

We tested the modelling prediction that γ-PGA overproduction would lead to global biofilm breakdown. To validate this gain of function, we monitored over time the progression of γ-PGA-overproducing biofilms and compared it with that of the wild-type strain. As in Fig. 4 above, we eliminated the starvation period to avoid the natural dispersion response. Consistent with the modelling prediction, the γ-PGA overproduction strain exhibited dramatic global biofilm disruption over time (Fig. 6c), which could not be accomplished simply by adding more glutamate (Extended Data Fig. 9). We observed large clusters of cells that appeared to be pushed outwards and then broke off from the main biofilm. This process globally continued over time such that new layers of cells were shed as the biofilm grew. By contrast, the wild-type biofilms exhibited neither dispersion nor break-up (Fig. 6a). For the γ-PGA overproduction biofilms, the dispersing clusters of cells were eventually flushed out of the chamber owing to directional media flow in the microfluidic device (Supplementary Video 5). By segmenting the images and colouring the connected clusters of cells, we visualized that the γ-PGA overproduction strain sloughs off several cell clusters over time (Fig. 6d,e). By contrast, the wild-type biofilm expanded over time but remained as one connected cluster with no dispersion (Fig. 6b,e). We were thus able to rupture the biofilm without nutrient starvation or drugs simply by overproducing γ-PGA.

## Discussion

The functional role of γ-PGA in biofilms has been a contentious point in the literature. It has even been suggested that γ-PGA plays no role in these bacterial communities^41,45^. However, by observing large biofilms with high resolution and over several days and subjecting them to transient starvation, we uncovered a previously unknown functional role for γ-PGA. Our results show that extracellular γ-PGA drives localized biofilm dispersion by pushing motile cells out of the biofilm. Biofilms are thus able to use an alternative dispersal mechanism that could, in principle, even operate in parallel to ECM degradation. Specifically, biofilms can use the swelling of extracellular γ-PGA to create osmotic forces that locally disperse motile cells in a directed manner. Interestingly, our mathematical modelling indicates that the presence of ECM can promote outward-directed motile cell dispersion by restricting isotropic γ-PGA-mediated movement. Concurrently, we show that γ-PGA-mediated dispersion can be osmotically suppressed and genetically controlled by either deleting or overproducing γ-PGA. The localized nature of dispersion shown in this work distinguishes it from the other osmotic features of biofilms, such as isotropic spreading, which helps expand the biofilm in all directions^46,47^. Importantly, we show that biofilms can be forced to break apart without the need for antibiotics or toxic chemicals by simply overproducing γ-PGA. These disrupted communities exhibit large spaces devoid of cells, suggesting that these biofilms would be easier to kill with drugs.

Given that global ECM degradation-based biofilm dispersion mechanisms have been described, what could be the benefit of localized dispersion? Previously described mechanisms of dispersion involve the secretion of extracellular enzymes that break down the matrix that holds the biofilm together, essentially ‘dissolving’ the entire biofilm^10,14,16,21^. By contrast, localized dispersion described here specifically enables motile cells to escape, whereas the matrix cells hold the rest of the biofilm together. Localized dispersion, specifically after transient starvation, could allow the biofilm to bet-hedge by releasing motile cells to colonize new environments while still keeping the rest of the community in its original location, perhaps to once again thrive as conditions improve.

Interestingly, diverse functions have been attributed to γ-PGA in other biological systems. For example, *Bacillus anthracis*, which causes anthrax, forms an extracellular γ-PGA capsule that helps its cells evade the host immune system^48,49^. Much research has investigated ways to control γ-PGA production in bacteria, such as through nutrient or electrical modulation^50–52^. From a conceptual and mechanistic perspective, the similarity between γ-PGA-driven biofilm dispersion and γ-PGA-driven nematocyst discharge in Cnidaria is rather striking (Fig. 6f,g). In both cases, the polyelectrolyte hydrogel nature of γ-PGA is used to drive expulsion. Interestingly, nematocysts exploit γ-PGA at the intracellular level, whereas biofilms use extracellular γ-PGA at the population level, demonstrating the versatility of this polymer. Insights gained from the biofilm dispersion phenomenon reported here could even help to better understand complex mammalian processes such as tumour metastasis. Tumours share features with bacterial biofilms^53–55^. For example, some tumour ECM components have hydrogel properties^1,56^. Our discovery of hydrogel-mediated cell expulsion in biofilms thus raises the intriguing possibility of bridging different areas of research for new control approaches^57,58^. Given that γ-PGA is produced by bacteria, archaea^59^ and eukaryotes, the biophysical properties of this polymer may help explain other capabilities of biological systems across the branches of life.

## Methods

### Bacterial strains

The *B. subtilis* strains used in this study are listed in Supplementary Table 1. The *capBCAE* deletion strain was created by amplifying the 5′ arm and 3′ arm fragments of the operon using PCR and assembling the fragments into a pER449 chromosomal integration vector using Gibson assembly. The *capBCAE* overexpression strain was created by amplifying the 5′ arm and 3′ arm fragments of the operon and assembling the fragments into an ECE174 chromosomal integration vector using Gibson assembly. The vector backbone contains the IPTG-inducible *hyperspank* promoter. All primers used are indicated in Supplementary Table 2, with overlapping regions for Gibson assembly indicated by lowercase letters. The *hag* deletion strain was derived from previous work^60^ with the P_*hag*_-*mCherry* motile cell reporter transformed into the strain. All transformations were performed using a standard protocol^61^. Where appropriate, growth media were supplemented with antibiotics at the following concentrations: 5 mg l^−1^ chloramphenicol, 9 mg l^−1^ neomycin, 300 mg l^−1^ spectinomycin, 6 mg l^−1^ tetracycline and 9 mg l^−1^ kanamycin.

### Growth conditions

Biofilms were grown on MSgg media (5 mM potassium phosphate buffer (pH 7.0), 100 mM 3-(*N*-morpholino)propanesulfonic acid (MOPS) buffer (pH 7.0, adjusted with NaOH), 2 mM MgCl_2_, 700 μM CaCl_2_, 50 μM MnCl_2_, 100 μM FeCl_3_, 1 μM ZnCl_2_, 2 μM thiamine HCl, 0.5% (v/v) glycerol and 0.5% (w/v) monosodium glutamate), previously reported to promote biofilm formation^24^. We used a version of MSgg with reduced buffer (1 mM MOPS) to enable the pH of the overall medium to be lowered to pH 4 (adjusted with HCl). This MSgg with 1 mM MOPS was previously used and shown to have no significant effects on *B. subtilis* biofilm growth^62^. The *hyperspank* promoter was fully induced using 1 mM IPTG.

### Growth in microfluidic device

We grew biofilms in commercially available CellASIC B04F microfluidic plates (Millipore-Sigma) following a previously published protocol^63^. The day before the experiment, we streaked the desired strains from −80 °C glycerol stocks onto Luria-Bertani (LB) agar plates with appropriate antibiotics and incubated the plates overnight at 37 °C. The following day, a single colony was inoculated into 3 ml LB liquid media and incubated with shaking at 37 °C for 5 h. Cells were pelleted at 2,100 × *g* for 1 min and resuspended in fresh MSgg. We loaded the cells into the microfluidic chamber following the manufacturer’s protocol. The B04F microfluidic plates have two pillars where cells can be trapped, providing an anchor for the biofilm to grow. After cell loading, we purged cells stuck under one of the two pillars in the growth chamber, leaving biofilm growth at only one of the two pillars. Cells were incubated at 30 °C with media being supplied at 1.5 psi.

To induce biofilm dispersion, we followed previous work^25–27^, which removed nutrients from biofilms and increased nutrients. We incubated biofilms in the microfluidic device using standard MSgg. After biofilms reached the desired size, typically around 30 h, we introduced MSgg media without glycerol or glutamate (MS), the sole carbon and nitrogen sources, respectively. After 16 h, we removed MS and flowed in standard MSgg for the remainder of the experiment, up to 72 h.

To aid the growth of the γ-PGA overexpression strain, we supplemented MSgg with 30 mM d-glutamic acid throughout the experiment. Given that γ-PGA is comprised of both l- and d-glutamic acid, adding d-glutamic acid to the medium provides a substrate for increased γ-PGA production.

### Time-lapse microscopy

Biofilms were observed using time-lapse microscopy with an Olympus IX83 inverted microscope (Evident Scientific) with a 10× objective (Olympus) and an X-Cite NOVEM light source (Excelitas Technologies). Images were taken at intervals of 30 s (Fig. 4) or 15 min (remaining figures) using an ORCA-Fusion BT camera (Hamamatsu).

### γ-PGA production measurement

To measure how much γ-PGA is produced, we adapted an established protocol^64^. First, we grew wild-type, Δ*capBCAE* and P_*hyp*_-*capBCAE* strains in liquid LB at 37 °C with shaking. After 4 h, all cultures were normalized to OD_600_ at 0.1 and resuspended in MSgg. We supplemented the P_*hyp*_-*capBCAE* strain with 30 mM d-glutamic acid as above. After 24 h growth at 37 °C with shaking, we centrifuged the culture at 2,100 × *g* for 10 min and kept only the supernatant. Next, we added 10 μM methylene blue to stain the γ-PGA. After 30 min incubation, we filtered the solution through a 0.22 μm filter. γ-PGA adsorbed the methylene blue but was too large to pass through the filter. We measured the methylene blue remaining in the filtrate using a NanoDrop 2000c spectrophotometer (Thermo Fisher Scientific) at 655 nm.

### Particle image velocimetry

To analyse the biofilm movies and track the movement of cells, we first spatially aligned the image stack using the MultiStackReg plug-in in FIJI (RRID:SCR_002285). Four images were taken for each biofilm, for each time point, one image for each quadrant. These four image stacks were stitched together using the BigStitcher plug-in for FIJI. To isolate the biofilm region for analysis, the region outside the biofilm was manually excluded. Next, we used the Particle Image Velocimetry (PIV) plug-in in ImageJ for each pair of successive timeframes to analyse the displacement of cells in the biofilm. To calculate the trajectory of cells across the movie, we analysed the PIV output in Python (RRID:SCR_008394). We generated the trajectory paths of each cell across time points in Python by discretely updating each cell’s position based on the PIV-derived displacement vectors. At intermediate positions on our PIV grid, we used SciPy^65^ to interpolate the displacement vectors.

### Particle tracking

To further analyse the movement of cells in a biofilm, we tracked visible features in the phase-contrast time-lapse images. We used FIJI^66^ to annotate the *X* and *Y* coordinates of these features for each time point, and the *X* and *Y* coordinates of the edge of the biofilm. These results were then visualized using Python. This technique was used for Extended Data Figs. 1 and 3.

### Fluorescent reporter analysis

To track the dispersing region, we monitored a fluorescent reporter for motile cells, P_*hag*_-*yfp*, using time-lapse microscopy. To visualize the dispersing region, we first binarized the fluorescence image using the Default threshold algorithm in FIJI, which allowed us to compare between different experiments and conditions. We saved the resulting image as a table of grey values using the Transform function in FIJI. We calculated and plotted the mean value for every pixel row of the image. This technique was used for Fig. 3b,d,f,h and Extended Data Fig. 7.

### γ-PGA overproduction analysis

To quantify the empty spaces inside the biofilm of the wild-type (WT) and γ-PGA overproduction strains, we first inverted and binarized the image using the Default threshold algorithm in FIJI. Areas where cells are located will have a value of 255, and areas devoid of cells will have a value of 0. We excluded the region outside the biofilm and the region under the cell trap for analysis by selecting regions of interest for these regions and reassigning these pixels NaN values using the changeValues function in FIJI. This exclusion will result in images with different total numbers of pixels, as biofilms are of different sizes. To compare between conditions, we divided the number of white pixels (value 255) or the number of black pixels (value 0) by the total number of pixels in the image.

### pH modulation analysis

To quantify the expansion and contraction of the biofilm owing to pH changes, we created a ‘difference movie’ by subtracting the pixel intensities of successive time points for each frame of the phase-contrast time-lapse movie. The pixel shifts shown by this difference movie were tracked using the Multi Measure function in FIJI.

### Cluster analysis

To compare dispersion from a WT biofilm versus a γ-PGA overproduction biofilm grown without starvation, we analysed the number of connected clusters for each biofilm. Using Python, we binarized the phase-contrast image to differentiate where cells are present or absent. Using the NetworkX library, we generated a network based on the maximal distance between biomass pixels. We quantified the number of connected clusters in the network for each biofilm using the KDTree function from SciPy^65^. To eliminate debris from the microfluidic device, we disregarded clusters below a threshold size.

### Statistics and data reproducibility

For all experiments, at least *n* = 3 independent biofilms were used unless otherwise noted. Independent experiments were performed on separate days with new media prepared for each experiment. The exact number of independent biofilms is indicated in the figure legends where the data appear. No tests of statistical significance were necessary and were thus not performed.

### Mathematical model

We show our biofilm system using a mathematical model based on partial differential equations for the densities of the two cellular populations (motile and non-motile), a hydrogel field and a nutrient field. The custom Python-based implementation of the simulations is publicly available at https://github.com/dsb-lab/dispersion. The parameter values used in the model are listed in Supplementary Table 3. We consider a two-dimensional continuum model for a growing biofilm composed of two cell phenotypes: a motile cell population with density ${\rho }_{m}\left({\bf{x}},t\right)$ and a non-motile (matrix) cell population with density ${\rho }_{n}\left({\bf{x}},t\right)$. Cell growth depends on the local availability of a nutrient field $C\left({\bf{x}},t\right)$. The motile population produces an extracellular hydrogel field of γ-PGA, denoted by $H\left({\bf{x}},t\right)$.

Biomass growth and γ-PGA hydrogel production act as local volumetric sources that generate an effective pressure field $P\left({\bf{x}},t\right)$, giving rise to a Darcy-type velocity field ${\bf{U}}\left({\bf{x}},t\right)$, which we assume to be unique for all the fields considered below^67^. The velocity field is therefore not divergence-free; instead, its divergence is determined by local biomass growth and γ-PGA production. In the absence of growth-induced volumetric sources, the velocity field becomes divergence-free.

The transport of ${\rho }_{m}$, ${\rho }_{n}$ and H is governed by advection by ${\bf{U}}$, supplemented by diffusive terms assuming a small miscibility between fields^68^. The nutrient field undergoes diffusion, consumption by the biofilm and replenishment from an external reservoir^67^.

The final model used in this paper was written in a non-dimensional form; the corresponding dimensional model and the non-dimensionalization procedure are detailed in the following sections.

### Dimensional model

We are working with a fluid chamber of small height, so we can consider a quasi-two-dimensional system. Then, let ${\bf{x}}\in \Omega \subset {{\mathbb{R}}}^{2}$ and t≥0. We define the following dimensional fields:

- ${\rho }_{m}\left({\bf{x}},t\right)$, ${\rho }_{n}\left({\bf{x}},t\right)$: densities of motile and non-motile (matrix) cells
- $H\left({\bf{x}},t\right)$: extracellular hydrogel (γ-PGA) concentration
- $$C\left({\bf{x}},t\right)$$: nutrient concentration
- $$P\left({\bf{x}},t\right)$$: effective pressure
- $${\bf{U}}\left({\bf{x}},t\right)$$: local velocity

We denote the total biomass by ${\rho }_{{\rm{tot}}}\left({\bf{x}},t\right)={\rho }_{m}\left({\bf{x}},t\right)+{\rho }_{n}\left({\bf{x}},t\right)$.

### Dimensional model—biomass dynamics

The dynamics of the cell densities are given by

$$\frac{\partial {\rho }_{m}\left({\bf{x}},t\right)}{\partial t}=\alpha \,{\rho }_{m}{\phi }_{\rho }(C)-\nabla \cdot ({\bf{U}}{\rho }_{m})+{D}_{\rho }{\nabla }^{2}{\rho }_{m},$$

$$\frac{\partial {\rho }_{n}\left({\bf{x}},t\right)}{\partial t}=\alpha \,{\rho }_{n}\,{\phi }_{\rho }\left(C\right)-\nabla \cdot \left({{\bf{U}}\rho }_{n}\right)+{D}_{\rho }{\nabla }^{2}{\rho }_{n}.$$

Here α denotes the maximum growth rate, the advective term $-\nabla \cdot \left({{\bf{U}}\rho }_{i}\right)$ describes the transport of biomass by the Darcy velocity field, and ${D}_{\rho }$ is a small effective diffusivity that captures a small miscibility. Cell growth is regulated by a saturating Michaelis–Menten-type response to nutrient availability (where *K*_*ρ*_ is the growth-rate half-maximal nutrient concentration):

$${\phi }_{\rho }\left(C\right)=\frac{C}{{K}_{\rho }+C}.$$

### Dimensional model—hydrogel dynamics

The extracellular polymer field evolves according to

$$\frac{\partial H\left({\bf{x}},t\right)}{\partial t}=\beta {\phi }_{h}\left(C\right){\rho }_{m}-\nabla \cdot ({\bf{U}}H)+{D}_{h}{\nabla }^{2}H,$$

where β is the γ-PGA production rate, the advective term $-\nabla \cdot ({\bf{U}}H)$ describes the transport of hydrogel by the Darcy velocity field, and ${D}_{h}$ is the γ-PGA diffusivity. γ-PGA production is activated by nutrient availability (where *K*_*h*_ is the γ-PGA half-maximal nutrient concentration) through

$${\phi }_{h}\left(C\right)=\frac{C}{{K}_{h}+C}.$$

We assume that polymer swelling saturates on a timescale much shorter than that of the biofilm growth and mechanical dynamics considered here. Consequently, we neglect explicit autocatalytic or self-swelling terms in the hydrogel dynamics. Under these assumptions, hydrogel dynamics is captured by a balance between production, advection and diffusion.

### Dimensional model—nutrient dynamics

The nutrient field obeys

$$\frac{\partial {\rm{C}}\left({\bf{x}},t\right)}{\partial t}=\eta q({\rho }_{{\rm{tot}}},H)\left({C}_{0}-C\right)-{\gamma }_{\rho }{\phi }_{\rho }\left(C\right){\rho }_{{\rm{tot}}}-{\gamma }_{h}{\phi }_{h}\left(C\right){\rho }_{m}+{D}_{c}{\nabla }^{2}C.$$

The first term in this equation represents nutrient replenishment from an external reservoir with concentration ${C}_{0}$ (the set point of the microfluidic nutrient flow), where *η* is the nutrient replenishment rate from external flow. This influx is modulated by biomass and hydrogel loading through the factor

$$q\left({\rho }_{{\rm{tot}}},H\right)=\frac{{K}_{c}}{{\rho }_{{\rm{tot}}}+H+{K}_{c}},$$

where *K*_*c*_ is the nutrient penetration coefficient, which reduces nutrient exchange in regions of high biomass or hydrogel density, thereby mimicking the limited penetration of the external nutrient flow into the biofilm.

We assume that nutrient transport occurs in a diffusion-dominated regime and therefore neglect the advective transport of nutrients by the biofilm velocity field. The parameter ${\gamma }_{\rho }$ denotes the nutrient consumption rate associated with cellular growth, whereas ${\gamma }_{h}$ accounts for nutrient consumption linked to γ-PGA production.

### Dimensional model—Darcy’s flow and pressure equation

We model biofilm mechanics using a Darcy-type closure,

$${\bf{U}}=-M\left({\rho }_{{\rm{tot}}},H\right)\,\nabla P,$$

where P is the pressure, calculated as discussed below, and the mobility is M, which depends on the biomass (total cell density) and on the hydrogel according to

$$M\left({\rho }_{{\rm{tot}}},H\right)={M}_{\rho }{\rho }_{{\rm{tot}}}+{M}_{{\rm{h}}}H+{M}_{0}\theta \left(1-{\rho }_{{\rm{tot}}}-H\right).$$

The parameter ${M}_{0}$ in this expression represents the mobility of the surrounding fluid; the ${M}_{\rho }$ and ${M}_{{\rm{h}}}$ terms account for the contributions of biomass and hydrogel to the effective mobility, respectively; and θ is the Heaviside function. In particular, we consider ${M}_{h} < {M}^{0}$, reflecting the polymeric, hydrogel-like nature of γ-PGA, which has a lower intrinsic mobility (that is, higher effective viscosity) than the surrounding fluid. Importantly, we have ${M}_{\rho } < {M}_{h}$, meaning that the hydrogel phase is more mobile than the densely packed biomass. Therefore, in regions initially dominated by dense biomass, the production and swelling of γ-PGA lead to an increase in local mobility. Biologically, this process is associated with a redistribution of biomass and a reduction in local cell density, effectively increasing the porosity of the biofilm. This results in an increase in effective large-scale mobility, consistent with our model. Biomass growth and γ-PGA production act as volumetric sources of the biofilm material. We define the total volumetric source as

$$Q=\alpha {\phi }_{\rho }\left(C\right){\rho }_{{\rm{tot}}}+\beta {\phi }_{h}\left(C\right){\rho }_{m},$$

where the first term on the right-hand side represents the contribution from cellular growth and the second term corresponds to hydrogel production.

These volumetric sources determine the divergence of the velocity field according to

$$\nabla \cdot {\bf{U}}=Q.$$

Substituting the Darcy relation ${\bf{U}}=-M\nabla P$ into this constraint yields a variable-coefficient Poisson equation for the pressure,

$$\nabla \cdot \left(M\nabla P\right)=-Q.$$

### Non-dimensionalization of the model

We now define non-dimensional variables as

$$\begin{array}{cccc} c=\frac{C}{{C}_{0}}, & {\tilde{\rho }}_{m}=\frac{{\rho }_{m}}{{\rho }_{0}}, & {\tilde{\rho }}_{n}=\frac{{\rho }_{n}}{{\rho }_{0}}, & h=\frac{H}{{H}_{\rho }}\end{array},$$

$$\begin{array}{cc} \widetilde{t}=\frac{t}{T}=\alpha t, & \widetilde{{\bf{x}}}=\frac{{\bf{x}}}{L}\end{array}.$$

We also define ${\widetilde{\rho }}_{{\rm{tot}}}={\widetilde{\rho }}_{m}+{\widetilde{\rho }}_{n}$. For notation simplicity, we will omit the tilde in cell density in what follows.

The scaling factors arise from characteristic scales for nutrient, biomass, hydrogel, time and length:

- Nutrient field: ${C}_{0}$ (reservoir concentration)
- Biomass: ${\rho }_{0}$ (packing density set by the initial conditions)
- Hydrogel: ${H}_{\rho }$ (hydrogel concentration equivalent in volume to ${\rho }_{0}$, set by the initial conditions)
- Time: $T=\frac{1}{\alpha }$ (growth timescale)
- Length: L (arbitrary reference scale)
  The scaling relations defined above lead us to the following dimensionless parameters:
- Diffusion
  $$\begin{array}{rcl} {\widetilde{D}}_{c}=\frac{{D}_{c}}{\alpha {L}^{2}}, & {\widetilde{D}}_{h}=\frac{{D}_{h}}{\alpha {L}^{2}}, & {\widetilde{D}}_{\rho }=\frac{{D}_{\rho }}{\alpha {L}^{2}}\end{array}.$$
- ECM production
  $$\widetilde{\beta }=\frac{\beta {\rho }_{0}\,}{\alpha {H}_{\rho }}.$$
- Nutrient consumption
  $$\begin{array}{cccc}{\tilde{\gamma }}_{\rho }=\frac{{\gamma }_{\rho }{\rho }_{0}}{\alpha {C}_{0}}, & {\tilde{\gamma }}_{h}=\frac{{\gamma }_{h}{\rho }_{0}}{\alpha {C}_{0}}, & \tilde{\eta }=\frac{\eta }{\alpha }, & {\tilde{K}}_{c}=\frac{Kc}{{\rho }_{0}}.\end{array}$$
- Nutrient influx
  $$\widetilde{q}\left({\rho }_{\mathrm{tot}},h\right)=\frac{{\widetilde{K}}_{c}}{{\rho }_{\mathrm{tot}}+\left(\frac{{H}_{\rho }}{{\rho }_{0}}\right)h+{\widetilde{K}}_{c}}=\frac{{\widetilde{K}}_{c}}{{\rho }_{\mathrm{tot}}+h+{\widetilde{K}}_{c}}.$$
  where we express hydrogel in the same normalized units as biomass (that is, ${H}_{\rho }\approx {\rho }_{0}$).
- Biomass and hydrogel growth factors
  $$\begin{array}{cc}{\widetilde{\phi }}_{\rho }\left(c\right)=\frac{\left({\chi }_{\rho }c\right)}{1+\left({\chi }_{\rho }c\right)},\, & {\widetilde{\phi }}_{h}\left(c\right)=\frac{\left({\chi }_{h}c\right)}{1+\left({\chi }_{h}c\right)},\end{array}$$
  with $${\chi }_{\rho }=\frac{{C}_{0}}{{K}_{\rho }}$$ and $${\chi }_{h}=\frac{{C}_{0}}{{K}_{h}}$$.
- Mobility
  $$\widetilde{M}\left({\rho }_{{\rm{tot}}},h\right)={\rho }_{{\rm{tot}}}+{\widetilde{M}}_{h}h+{\widetilde{M}}_{0}\theta \left(1-{\rho }_{{\rm{tot}}}-h\right),$$
  where ${\widetilde{M}}_{0}={M}_{0}/{M}_{\rho }$ represents the mobility of the surrounding fluid relative to the biomass scale and ${\widetilde{M}}_{h}={M}_{h}/{M}_{\rho }$ quantifies the relative (dimensionless) mobility of the γ-PGA hydrogel.
- Darcy’s law
  $${\bf{u}}=-\widetilde{M}\nabla p,$$
  where $\widetilde{\mu }$ is defined above, and the dimensionless pressure is
  $$\begin{array}{cc}\displaystyle p=\frac{P}{\widetilde{P}}, & \mathrm{with}\,\widetilde{P}=\frac{{L}^{2}\alpha }{{M}_{\rho }}\end{array}.$$

The non-dimensional pressure can be calculated via

$$\nabla \cdot \left(\widetilde{M}\nabla p\right)=-{Q}_{{\rm{tot}}},$$

where

$${Q}_{\mathrm{tot}}={\rho }_{m}{\widetilde{\phi }}_{\rho }\left(c\right)+{\rho }_{n}{\widetilde{\phi }}_{\rho }\left(c\right)+\beta {\widetilde{\phi }}_{h}\left(c\right){\rho }_{m}.$$

#### Full non-dimensional model

With these rescaling and dimensionless parameters, and dropping the remaining tildes for readability, the final non-dimensional model is

$${\partial }_{t}{\rho }_{m}={\rho }_{m}{\phi }_{\rho }\left(c\right)-\nabla \cdot \left({\rho }_{m}{\bf{u}}\right)+{D}_{\rho }{\nabla }^{2}{\rho }_{m},$$

$${\partial }_{t}{\rho }_{n}={\rho }_{n}{\phi }_{\rho }\left(c\right)-\nabla \cdot \left({\rho }_{n}{\bf{u}}\right)+{D}_{\rho }{\nabla }^{2}{\rho }_{n},$$

$${\partial }_{t}h=\beta {\phi }_{h}\left(c\right){\rho }_{m}-\nabla \cdot \left(h{\bf{u}}\right)+{D}_{h}{\nabla }^{2}h,$$

$${\partial }_{t}c=\eta q({\rho }_{{\rm{tot}}},h)\left(1-c\right)-{\gamma }_{\rho }{\phi }_{\rho }\left(c\right){\rho }_{{\rm{tot}}}-{\gamma }_{h}{\phi }_{h}\left(c\right){\rho }_{m}+{D}_{c}{\nabla }^{2}c.$$

We note that parameters (for example, *α*, *C*_0_ and *M*_*ρ*_) are used as reference scales in the non-dimensionalization procedure and are therefore absorbed into the definition of the dimensionless variables. Consequently, they do not appear as independent parameters in the final system, which is equivalent to setting them equal to 1.

### γ-PGA overproduction in the model

To model γ-PGA overproduction, we introduce an overproduction factor ${\eta }_{{\rm{OE}}}\ge 1$ that multiplies the effective polymer production rate. In the dimensionless formulation, this is implemented by rescaling the γ-PGA production parameter β as

$$\beta \to \,{\eta }_{\mathrm{OE}}\beta .$$

As all cells in the γ-PGA overproduction strain contain the inducible *capBCAE* operon, we consider that matrix cells are also able to produce γ-PGA in this strain. We assume this production to be heterogeneous. Gene expression in bacterial populations is inherently heterogeneous, even in phenotypically defined subpopulations. In *B. subtilis* biofilms, this heterogeneity extends to the matrix-producing fraction and persists under inducible expression^69–71^. We represent the fraction of matrix cells that produce γ-PGA by a third cell-density field, which has the same effect on the hydrogel field as the motile cells, but is made up of non-motile cells.

To model supplementation with d-glutamic acid, we introduce a rescaling factor ${\eta }_{C}$ for the nutrient field in the dimensional set-point concentration ${C}_{0}$. Guided by the experimental design, we assume that the added d-glutamic acid only increases the ability of cells to produce γ-PGA, without modifying the growth terms directly. After the non-dimensionalization, this supplementation leads to the following parameter rescaling:

$${\chi }_{h}\to {\eta }_{C}{\chi }_{h},$$

$${\gamma }_{h}\to {\eta }_{{OE}}{\gamma }_{h}/{\eta }_{C}.$$

We note that the overproduction factor ${\eta }_{{\rm{OE}}}$ also affects the γ-PGA-associated consumption term parametrized by ${\gamma }_{h}$, as polymer secretion requires metabolic resources.

### Initial conditions, boundary conditions and numerical integration parameters

Initial biomass is prescribed as a compact colony centred within the simulation domain, with geometric perturbations at the periphery to break radial symmetry. The simulation domain is a two-dimensional square $\left[0,{L}_{x}]\times [0,{L}_{y}\right]$ with ${L}_{x}={L}_{y}=20$ (dimensionless units), discretized on a uniform grid of ${N}_{x}={N}_{y}=1,024$ cells. The pressure field is solved using Dirichlet boundary conditions, P=0, at the domain boundary. For the remaining fields, Dirichlet boundary conditions are imposed consistently with the numerical implementation: ${\rho }_{m}={\rho }_{n}=h=0$ and c=1 at the boundary. Advection terms are discretized using a first-order upwind scheme, whereas diffusive and pressure operators are approximated using finite differences on the non-periodic grid.

#### Initial biomass geometry and phenotype partition

The two phenotypes are initialized in distinct subregions: a core colony region with a protruding plume-like region and a periphery.

- A baseline circular colony of radius ${R}_{{\rm{bio}}}=1.5$ is placed at the domain centre $\left({x}_{0},{y}_{0})=({L}_{x}/2,{L}_{y}/2\right)$.
- The biofilm interface is perturbed by an angular Fourier noise constructed from a finite number of modes (k=1,…,6) with random phases. This produces a perturbed radius
  $${R}_{{\rm{bio}}}(\theta )={R}_{{\rm{bio}}}\left[1+{\varepsilon }_{{\rm{core}}}\eta (\theta )\right],$$
  with ${\varepsilon }_{{\rm{core}}}=0.08$, where $\eta (\theta )$ is a normalized sum of cosine modes. This perturbation breaks the circular symmetry and seeds morphological heterogeneity at t=0.
- The matrix phenotype ${\rho }_{n}$ is first assigned to the biofilm region and bounded in [0, 1].
- A plume region is then constructed and assigned to the motile phenotype ${\rho }_{m}$. The plume is defined as the union of the following:

1. a perturbed circular cap of radius ${R}_{{\rm{plume}}}=0.5\,$(with perturbation amplitude 0.10), and
2. a tapered, cone-like extension of length ${L}_{{\rm{plume}}}=3/2\,{R}_{{\rm{plume}}}$ and base half-width ${R}_{{\rm{core}}}=0.2$, whose width decreases with distance from the base.

Inside the plume mask, ${\rho }_{n}$ is set to 0 and ${\rho }_{m}$ is set to 1. In that way, we are able to mimic one of the characteristic branches that are formed by motile cells during biofilm maturation.

In the overproduction case, add N=50 randomly located ‘spots’ of γ-PGA-producing matrix cells (see ‘γ-PGA overproduction in the model’) in the matrix region.

To avoid sharp interfaces, the initial fields are regularized using a smooth hyperbolic tangent profile with characteristic width Δx.

#### Nutrient and hydrogel initialization

The nutrient is initialized uniformly at 0, c(x,0)=0, and the γ-PGA hydrogel is initialized as h(x,0)=0 everywhere.

### Reporting summary

Further information on research design is available in the Nature Portfolio Reporting Summary linked to this article.

## Supplementary information


Supplementary InformationSupplementary Tables 1–3.
Reporting Summary
Supplementary Video 1Wild-type biofilm undergoing localized dispersion. Time indicated in hours. Scale bar, 200 μm.
Supplementary Video 2Wild-type biofilm undergoing localized dispersion with motile and matrix cell type reporters. Phase-contrast image is on the left. Motile cell type fluorescent reporter (middle) is in orange. Matrix cell type fluorescent reporter (right) is in green. Time indicated in hours. Scale bar, 100 μm.
Supplementary Video 3Mathematical model recapitulates localized dispersion. Motile cells are in orange, and matrix cells are in green.
Supplementary Video 4Mathematical model predicts biofilm break-up in a γ-PGA overproduction biofilm. Motile cells are in orange, and matrix cells are in green.
Supplementary Video 5γ-PGA overproduction forces biofilm break-up. Direction of media flow in the microfluidic device is indicated by the yellow arrow. Time indicated in hours. Scale bar, 100 μm.


## Data Availability

The data that support the findings of this study are available from the corresponding author upon request. Requests should be made to G.M.S., and a response can be expected within 2 weeks of receiving the request.

## Code availability

The code that implements our mathematical model is available at https://github.com/dsb-lab/dispersion.
