# Supplementary material for: Self-generated hydrogel ejects bacterial cells for localized biofilm dispersion
Source: Nat Microbiol. 2026 Jul 7;11(9):2423–35. doi: 10.1038/s41564-026-02413-4 (PMC13524836; doi:10.1038/s41564-026-02413-4)
Supplement: Supplementary file 1 [file 41564_2026_2413_MOESM1_ESM.pdf]

nature microbiology

Supplementary information

https://doi.org/10.1038/s41564-026-02413-4

Self-generated hydrogel ejects bacterial cells for localized biofilm dispersion

In the format provided by the authors and unedited

Supplementary Information

Supplementary Table 1. Bacterial strains used in this study.

Bacterial strain	Source
B. subtilis NCIB 3610 amyE::PyqxM-mCherry, sacA::Phag-yfp	Comerci et al.[31]
B. subtilis NCIB 3610 capBCAE::Neo, amyE::PyqxM-mCherry, sacA::Phag-yfp	This paper
B. subtilis NCIB 3610 amyE::Phag-mCherry, sacA::Phyperspank-capBCAE	This paper
B. subtilis NCIB 3610 hag::CAT, amyE::Phag-mCherry	This paper
B. subtilis NCIB 3610 sacA::PcapB-yfp	This paper
B. subtilis NCIB 3610 epsH::tet	Branda et al.[41]
B. subtilis NCIB 3610 tasA::kan	Vlamakis et al.[30]

Supplementary Table 2. Primers used in this study.

Primer name	Primer sequence
GS3013	gctgataccgctcgccGCGTTCTGTCCAGAAAAACAACTGG
GS3014	ccttcctccaaatcagacaGACTAAGGAGGTTCGAGATGAAAACGC
GS3015	gacagcttatcatcggcaCCAGCAGCAGGAAGAACCGG
GS3016	cttaactatgcggcatcagaGGACACCGATATATTTTTCGGCCTCC
GS2964	cttaaggaggaaagtcacattATGTGGTTACTCATTATAGCCTGTGCTGTC
GS2965	gtaatccgcccgacggtatcTTATTTATTGGCGTTTACCGGTTCTTCCTG

Supplementary Table 3. Parameter values used in the model. The behavior of the model is governed by these dimensionless control parameters.

Parameter	Description	Value
**Nutrient uptake and regulation**		
χ_ρ	Cell growth activation by nutrient availability	1000
χ_h	γ-PGA production activation by nutrient availability	100
**Transport parameters**		
D_c	Nutrient diffusivity	1
D_ρ	Biomass diffusivity	10^{-2}
D_h	γ-PGA diffusivity	10^{-2}
**Reaction and production rates**		
β	γ-PGA production rate	3
γ_ρ	Nutrient consumption by biomass growth	5
γ_h	Nutrient consumption associated with γ-PGA production	50
η	Nutrient replenishment rate from the external flow	10^4
**Mechanical parameters**		
M_0	Relative mobility of the surrounding fluid	100
M_h	Relative mobility of the γ-PGA hydrogel	50
**Nutrient penetration parameter**		
K_c	Biomass/γ-PGA loading scale controlling nutrient replenishment	10^{-3}
**Overproduction parameter**		
η_{OE}	γ-PGA overproduction factor	4
η_{C}	D-glutamic acid supplementation factor	2
